# Global Analysis of Apicomplexan Protein S-Acyl Transferases Reveals an Enzyme Essential for Invasion

**DOI:** 10.1111/tra.12081

**Published:** 2013-05-29

**Authors:** Karine Frénal, Chwen L Tay, Christina Mueller, Ellen S Bushell, Yonggen Jia, Arnault Graindorge, Oliver Billker, Julian C Rayner, Dominique Soldati-Favre

**Affiliations:** 1 Department of Microbiology and Molecular Medicine, CMU, University of GenevaRue Michel-Servet 1, CH-1211, Geneva 4, Switzerland; 2 Malaria Programme, Wellcome Trust Sanger Institute, Wellcome Trust Genome Campus, HinxtonCambridge, CB10 1SA, UK

**Keywords:** Apicomplexa, egress, invasion, palmitoyl acyl transferase, palmitoylation, *Plasmodium berghei*, *Plasmodium falciparum*, rhoptry organelle, *Toxoplasma gondii*

## Abstract

The advent of techniques to study palmitoylation on a whole proteome scale has revealed that it is an important reversible modification that plays a role in regulating multiple biological processes. Palmitoylation can control the affinity of a protein for lipid membranes, which allows it to impact protein trafficking, stability, folding, signalling and interactions. The publication of the palmitome of the schizont stage of *Plasmodium falciparum* implicated a role for palmitoylation in host cell invasion, protein export and organelle biogenesis. However, nothing is known so far about the repertoire of protein S-acyl transferases (PATs) that catalyse this modification in Apicomplexa. We undertook a comprehensive analysis of the repertoire of Asp-His-His-Cys cysteine-rich domain (DHHC-CRD) PAT family in *Toxoplasma gondii* and *Plasmodium berghei* by assessing their localization and essentiality. Unlike functional redundancies reported in other eukaryotes, some apicomplexan-specific DHHCs are essential for parasite growth, and several are targeted to organelles unique to this phylum. Of particular interest is DHHC7, which localizes to rhoptry organelles in all parasites tested, including the major human pathogen *P. falciparum*. TgDHHC7 interferes with the localization of the rhoptry palmitoylated protein TgARO and affects the apical positioning of the rhoptry organelles. This PAT has a major impact on *T. gondii* host cell invasion, but not on the parasite’s ability to egress.

The phylum Apicomplexa includes important obligate intracellular pathogens responsible for severe diseases affecting humans and livestock. *Plasmodium falciparum* is responsible for the most severe form of malaria causing mortality and morbidity in humans, whereas *Toxoplasma gondii* causes toxoplasmosis, which affects human and warm-blooded animals. As members of the genus Alveolata, the apicomplexans possess a pellicle composed of the plasma membrane (PM) and the inner membrane complex (IMC) formed by flattened cisternae underlining the PM and connected to the subpellicular microtubules. The IMC is interconnected with the parasite cytoskeleton and plays a fundamental role in motility and cytokinesis 2. Apicomplexans have adopted an intracellular life style and rely on a common active mode of host cell entry that provides them a unique opportunity to infect a broad range of cell types without stimulating the host cell defence mechanisms 3. Host cell entry is initiated when specialized organelles called micronemes and rhoptries secrete their contents 1. The invasive stages exhibit an unusual form of gliding motility that involves the concerted action of at least one myosin motor, regulators of actin dynamics, adhesins and proteases 1. The gliding machinery termed ‘glideosome’ is a unique attribute of the phylum Apicomplexa, which is crucial to actively cross non-permissive biological barriers and to penetrate into and egress from host cells 4.

Many of the proteins involved in glideosome assembly and function, associated to the IMC and implicated in organelle biogenesis appear to be post-translationally modified by acylation. Protein palmitoylation in particular is emerging as a fundamental, dynamic and widespread post-translational mechanism that controls transport, properties and activity of proteins across eukaryotes. Unlike other irreversible lipid modifications such as myristoylation and prenylation, the addition of a 16-carbon saturated palmitate group to the sulphydryl group of a cysteine to form a hydroxylamine-sensitive thioester linkage is a reversible modification. This constitutes a fast and dynamic mechanism to spatiotemporally control protein function by impacting reversibly on protein trafficking, stability and clustering 5. While palmitoylation frequently facilitates membrane association of a soluble protein by the addition of a hydrophobic anchor, this modification also occurs on transmembrane proteins, involving other effects such as structural conformation changes, protein–protein interactions or the clustering to specific lipid domains leading, for example, to assembly of signalling complexes 6.

The enzymes mediating transfer of palmitate from palmitoyl-CoA to a protein substrate were first identified in *Saccharomyces cerevisiae* 7,8 and subsequently in mammals 9. Protein S-acyl transferases (PATs) belong to the Asp-His-His-Cys (DHHC) family of proteins that exhibit a catalytic Asp-His-His-Cys conserved motif located within a cysteine-rich domain (CRD) and frequently between two transmembrane regions facing the cytosol 8–10. Substrate recognition and catalysis occur after the protein substrates have associated with membrane via another lipidation 11. In yeast, three PATs are localized in the ER, two in the Golgi, one at the PM and one at the vacuole 12, and they can also be divided into three categories depending on their structure: ankyrin-repeat containing, heterodimeric or monomeric 13. Deletion and overexpression studies in yeast showed redundancy in PAT function 12. Knowledge about mechanisms that govern their localization, substrate specificity or regulation is incomplete.

Palmitoylation in apicomplexan parasites has only recently become a subject of study, instituted primarily by studies of the gliding-associated protein GAP45, which is critical to host cell invasion across the phylum 14. Functional investigation revealed that palmitoylation of TgGAP45 is essential for recruiting the motor to the IMC and for maintenance of the pellicle integrity 15. *Plasmodium falciparum* calpain is a cysteine protease required for cell cycle progression and its acylation is critical for the shuffling of the protease between the nucleus and the ER 16,17. Other components of the invasion process also appear to be regulated by palmitoylation, such as the armadillo repeats containing protein (Pf/TgARO), which is localized at the periphery of the secretory rhoptry organelles via palmitoylation 18. More invasion-associated parasite proteins are predicted to be palmitoylated including some implicated in signalling such as some members of the calcium-dependent protein kinases 19,20, additional components of the glideosome GAP70, MLC1 15 and proteins associated to the IMC such as the family of IMC subcompartment proteins ISPs 21,22 and the filament-like alveolins 23. Recently, the report of the palmitome of *P. falciparum* revealed more than 400 putative palmitoylated proteins in the schizont stage (the intraerythrocytic stage when parasite multiplication occurs, 42–48 h post-invasion and results from multiple fissions of the nucleus followed by cellular segmentation) 24.

Palmitoylation clearly plays a central role in the biology of apicomplexan parasites in general and regulates host cell invasion in particular, but nothing is currently known about the parasite enzymes responsible for this modification. To gain insights into the importance of palmitoylation for parasitism by the Apicomplexa, we have determined the repertoire of DHHC-CRD S-acyl transferase protein family as putative candidates for PATs and determined their subcellular distribution and essentiality in two parasites of the phylum. Sixteen of 18 *T. gondii* DHHC family members are expressed in tachyzoites and 5 of these appeared to be essential for survival. In *Plasmodium berghei* there was evidence to suggest that 2 of 11 DHHCs tested may be essential in blood stages, and as in *T. gondii*, individual *P. berghei* DHHCs were localized to different intracellular organelles. Notably, DHHC7 was localized to the rhoptries across the phylum, including in the human pathogen *P. falciparum*. Given that rhoptries are specialized apicomplexan specific organelles that crucially contribute to invasion and establishment of infection by subversion of host cellular function 25, we chose to dissect the function of TgDHHC7 further. The Cre recombinase-dependent conditional excision of *TgDHHC7* gene demonstrates that this protein acts as PAT for the rhoptry acylated TgARO and impacts the apical positioning of the secretory organelle, a prerequisite for host cell invasion by the parasites but not egress from infected cells.

## Results

### Repertoire of DHHC motif containing proteins in Apicomplexa

A BLAST search across available apicomplexan genomes was performed in order to identify the repertoire of DHHC motif containing proteins using the DHHC-CRD of *S. cerevisiae* Erf2 as a query 7. This analysis revealed a large repertoire of putative PATs in Apicomplexa, with the largest family composed of 18 proteins in *T. gondii* and 17 members in the closely related pathogen *Neospora caninum*. *Plasmodium falciparum* and *P. berghei* possess 12 and 11 genes, respectively, while *Theileria parva* and *Babesia bovis* have 9 and 8 genes, respectively. The more distant *Cryptosporidium* species contain 10 genes, whereas 6 genes have been identified in the partially annotated *Eimeria tenella* genome (Figure S6, Supporting Information). By contrast the *S. cerevisiae* contain 7 PAT genes, while humans possess 23 8,10.

For further experimental analysis in *T. gondii*, the complete sequence of each ORF was needed. Therefore, all the genes for which no EST data were available 26 to determine the full-length sequence were experimentally annotated by polymerase chain reaction (PCR) amplification from tachyzoite cDNAs and the products sequenced. These annotations, confirmed by the RNAseq information recently available on ToxoDB 26, were submitted to NCBI GenBank and the gene accession numbers are indicated in Table 1. Two of the genes coding for DHHCs, *TgDHHC10* and *TgDHHC18*, failed to be amplified in tachyzoites, indicating that these genes are stage specific. In contrast to the other DHHC genes, the expression profiles of *TgDHHC10* and *TgDHHC18* were flat and low throughout the cell cycle 27 (Figure S1A), and no active promoter 28 or transcripts have been detected. In addition, EST data for TgDHHC10 suggest that the protein is expressed in the oocyst stage, whereas TgDHHC18 might be expressed in bradyzoites. *Plasmodium* DHHCs also showed some evidence of stage-specific expression, with strong evidence for gametocyte stage expression for *PfDHHC6* and *PfDHHC10* 29.

**Table 1:** Repertoire of DHHC-containing PATs in *T. gondii*

Name	ToxoDB^a^ accession number	NCBI protein accession number	Localization^b^	Essentiality^b^	Motifs
TgDHHC1	TGME49_250870	AFW99801	Golgi	No	−PG/TTxE + Kxx
TgDHHC2	TGME49_278850	AFW99802	IMC	Yes	DPG/TTxE
TgDHHC3	TGME49_217870	AFW99803	ER/vesicles	No	DPG/TTxE
TgDHHC4	TGME49_213550	AFW99804	Plasma membrane	No	DPG/TTxE
TgDHHC5	TGME49_224290	AFW99805	Golgi	Yes	DPG/TTxE
TgDHHC6	TGME49_224310	AFW99806	Golgi	No	DPG/TTxE
TgDHHC7	TGME49_252200	AFW99807	Rhoptries	Yes	−PG/TTxE
TgDHHC8	TGME49_255650	AFW99808	ER/vesicles	No	NPG/TTxE + KKxx
TgDHHC9	TGME49_269150	AFW99809	Golgi	Yes	DPG/TTxxE
TgDHHC10	TGME49_301370	EEB04519	n.d.	n.d.	−/STxE
TgDHHC11	TGME49_284170	AFW99810	Golgi	No	DPG/TTxxE
TgDHHC12	TGME49_29160	AFW99811	Golgi	No	DPG/−
TgDHHC13	TGME49_249380	AFW99812	Plasma membrane	No	DP−/TTxE
TgDHHC14	TGME49_293730	AFW99813	IMC (cap excluded)	Yes	D-G/TxxE
TgDHHC15	TGME49_293220	AFW99814	Golgi	No	DPG/TTxE
TgDHHC16	TGME49_266940	AFW99815	ER/nuclear membrane	No	DPG/TTxE
TgDHHC17	TGME49_272320	AFW99816	Golgi	No	DPG/TxxE + Kxx
TgDHHC18	TGME49_246650	EEA99912.1	n.d.	n.d.	NPG/TT--

n.d., not determined.

a ToxoDB version 8.0 36.

b Based on this study for the tachyzoite stage.

**Figure 1:**
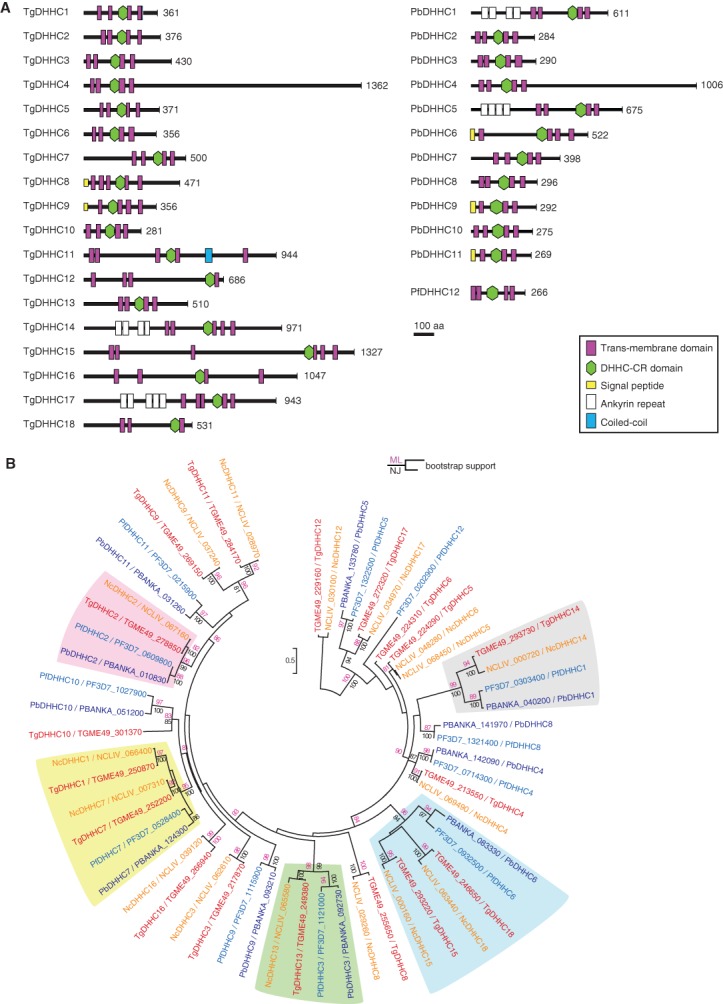
Repertoire of DHHC-containing proteins in *T. gondii* and *P. berghei* and their phylogenetic relationship. A) Schematic representation of the primary structure of the DHHCs highlighting their different domains; the domains have been searched with SMART (http://smart.embl-heidelberg.de) 30,31, the transmembrane domain predictions have been performed with TMHMM 2.0 server (http://www.cbs.dtu.dk/services/TMHMM) 32 or with TMPred server (http://www.ch.embnet.org/software/TMPRED_form.html) and the signal peptides have been predicted with signalP 4.0 server (http://www.cbs.dtu.dk/services/SignalP) 33. Phylogenetic tree of the *T. gondii*, *N. caninum*, *P. falciparum* and *P. berghei* DHHC family of proteins based on NJ 34 distance analysis on one hand and ML 35 on the other hand. Only nodes supported by a bootstrap value >80 are indicated and values >95 were considered as significant allowing to cluster sequences (coloured boxes). Protein numbers are given according to the EuPathDB website 36. See also Figure S1 for the phylogenetic tree across available Apicomplexa genomes and Figure S6 for the multiple sequences alignment used to compute the phylogenetic trees.

A bioinformatic analysis was carried out to identify the domains and motifs present on the predicted amino acid sequences. As expected, all *T. gondii*, *P. falciparum* and *P. berghei* putative PATs are polytopic proteins with at least four transmembrane domains and exhibit a conserved DHHC-CRD (Figure 1A and Tables 1 and 2). In each repertoire, except *N. caninum* and *E. tenella*, one protein harbours a DHYC amino acid motif, rather than the canonical DHHC, which is known to be functional in the yeast Akr1 protein 8. The two short motifs DPG and NxTTxE 37 that are usually conserved in the DHHC family are also present in most of the apicomplexan putative PATs (Tables 1 and 2) as well as predicted palmitoylation sites, and in each repertoire two putative PATs have ankyrin repeats in their N-terminal domains. Two proteins in *T. gondii* have predicted signal peptides 33 and three have predicted signal peptides in *P. berghei* and *P. falciparum* (Figure 1A). However, despite this signal, the DHHC domain is still predicted to face the cytoplasm. Three proteins might be targeted to the endoplasmic reticulum (ER) in *T. gondii* because they present a lysine-based sorting motif at their extreme C-terminus, a KKXX for TgDHHC8 and a KXX motif for TgDHHC1 and TgDHHC17 38.

**Table 2:** Repertoire of DHHC-containing PATs in *P. berghei*

Name	PasmoDB P.f. accession number	PasmoDB P.b. accession number	Closest homologue in *T. gondii*^b^	Pb localization^c^	Pb essentiality^d^	Motifs
PfDHHC1	PF3D7_0303400	PBANKA_040200	TgDHHC14	n.s.	n.d.	NPG/TFxE
PfDHHC2	PF3D7_0609800	PBANKA_010830	TgDHHC2	n.s.	n.d.	DPG/TTxE
PfDHHC3	PF3D7_1121000	PBANKA_092730	TgDHHC13	IMC	No	DPL/TTxE
PfDHHC4	PF3D7_0714300	PBANKA_142090	TgDHHC4	n.s.	n.d.	DPG/TTxE
PfDHHC5	PF3D7_1322500	PBANKA_133780	TgDHHC17	ER	No	−PG/TLxE + Kxx
PfDHHC6	PF3D7_0932500	PBANKA_083330	TgDHHC15/18	n.s.	No	–/TTxE
PfDHHC7	PF3D7_0528400	PBANKA_124300	TgDHHC1/7	Rhoptry	No	−PG/TTxE
PfDHHC8	PF3D7_1321400	PBANKA_141970	–	Punctate, not Golgi	n.d.	DPG/TTxE
PfDHHC9	PF3D7_1115900	PBANKA_093210	–	IMC	No	NPG/TTxxE
PfDHHC10	PF3D7_1027900	PBANKA_051200	TgDHHC10	n.s.	No	SPG/TTxE + Kxx
PfDHHC11	PF3D7_0215900	PBANKA_031260	–	n.s.	No	−PG/– + Kxx
PfDHHC12	PF3D7_0202900	–	–	n.d.	n.d.	DPG/TxxE

n.d., not determined; n.s., no signal; P.f., P. falciparum; P.b.: P. berghei.

^a^ PlasmoDB version 9.2 36.

b Based on the phylogenetic analyses of this study.

c Based on this study.

d Based on this study for the intraerythrocytic stages.

### The evolutionary relationship between the DHHCs in the phylum Apicomplexa

To understand the relationship between the different DHHC-containing proteins and their conservation across the phylum, a phylogenetic analysis was performed using the neighbour-joining (NJ) 34 and maximum likelihood (ML) 35 methods. A multiple alignment of the conserved DHHC-CRD, the only domain to be conserved in all DHHCs, and the DPG and NxTTxE motifs (≈77 amino acids) were used to build a phylogenetic tree including *T. gondii*, *N. caninum*, *P. falciparum* and *P. berghei* sequences (Figure 1B) as well as across the Apicomplexa (Figures S1B and S6). For five putative PATs, there are clear orthologues between the two organisms (Figure 1B and Table 2). TgDHHC2 corresponds to PBANKA_010830 and PF3D7_0609800 (Pb/PfDHHC2), while TgDHHC13 corresponds to PBANKA_092730 and PF3D_1121000 (Pb/PfDHHC3). The ankyrin repeats containing proteins are conserved: TgDHHC14 is a close homologue of PBANKA_040200 and PF3D7_0303400 (Pb/PfDHHC1). For two of the other *Plasmodium* DHHCs, there are two related *Toxoplasma* and *Neospora* genes that are not arising from gene duplication: TgDHHC1 and TgDHHC7 group with PBANKA_124300 and PF3D7_0528400 (Pb/PfDHHC7), whereas TgDHHC15 and TgDHHC18 group with PBANKA_083330 and PF3D7_0932500 (Pb/PfDHHC6) (Figure 1B and Table 2). For the other PbDHHCs, there are related TgDHHCs, but it was not possible to assign specific homologues because of the low bootstrap values. The *P. berghei* and *P. falciparum* genes all clearly organized into orthologous pairs, with the exception of PfDHHC12, which is absent from all rodent *Plasmodium* species, although homologues are present in other human *Plasmodium* species. Indeed, PfDHHC12 seems to be highly specific of the human *Plasmodium* species because it also does not group with any other apicomplexan DHHC-containing proteins (Figure S1B). Only six significant clusters are found across the entire phylum (Figure S1B). TgDHHC2, TgDHHC13 and TgDHHC14 are found in all Apicomplexa included in the analysis. The DHYC-containing proteins group together with the *T. gondii* one, TgDHHC10, being a little more distant. TgDHHC7 and TgDHHC1 fall into a tightly conserved cluster, which includes a single DHHC gene in all other apicomplexan species, and finally the cluster including TgDHHC15, TgDHHC18, PbDHHC6 and PfDHHC6 also include one *Eimeria* and *Cryptosporidium* sequences. Based on these homology patterns, *Plasmodium* DHHC gene numbers were coordinated with their closest *T. gondii* homologue wherever possible (Table2).

### Expression of the DHHCs in *T. gondii* and *P. berghei*

**Figure 2:**
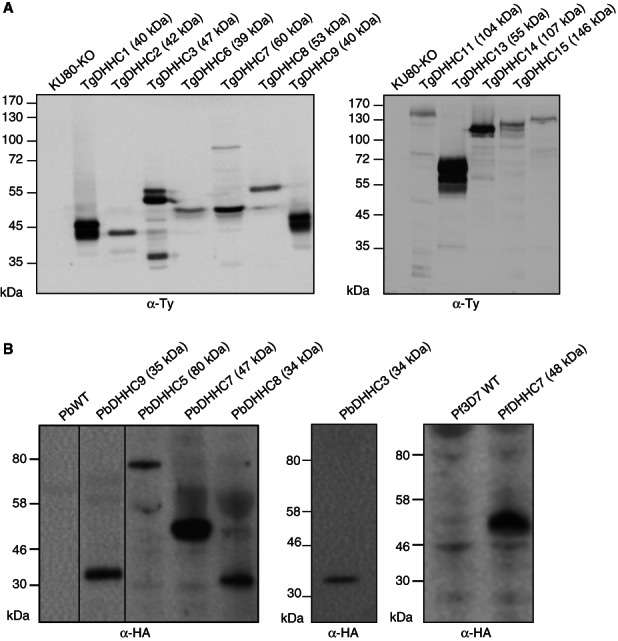
Expression of the putative PATs. Western blot performed on total protein extracts from *T. gondii* tachyzoite expressing endogenous Ty-tagged DHHCs (A) and *P. berghei* or *P. falciparum* schizonts expressing endogenous HA-tagged DHHCs (B). The membranes have been probed with anti-Ty and anti-HA antibodies, respectively. The expected sizes are indicated in brackets. See also Figure S2 for the generation of the tagged strains in both *T. gondii* and *P. berghei*.

**Figure 3:**
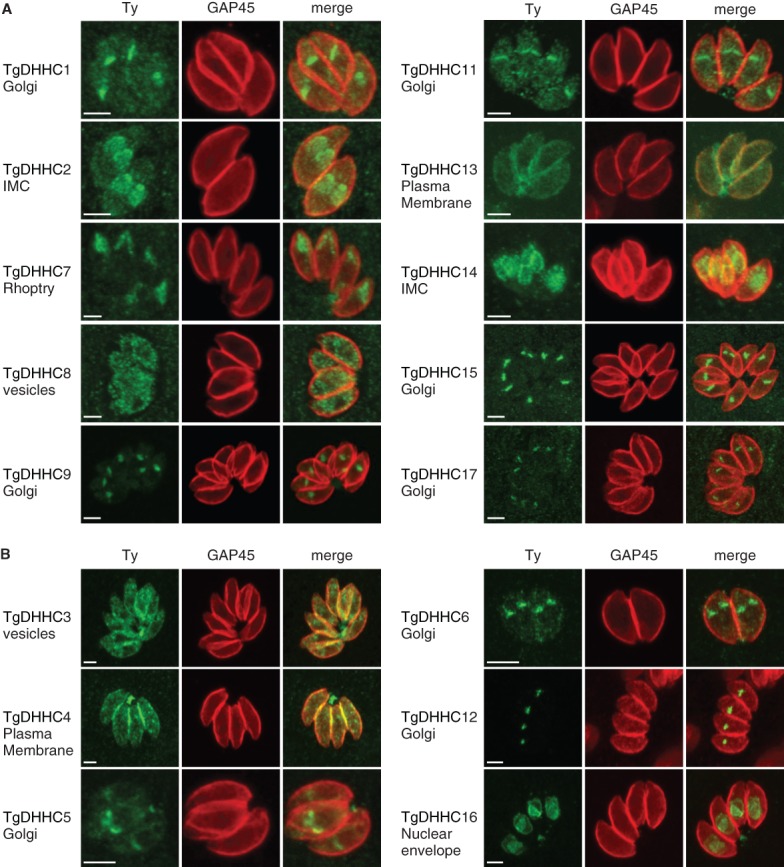
Localization of the DHHC-containing proteins in *T. gondii.* A) Subcellular localization of the endogenous putative TgPATs tagged at their C-terminal extremity by a knockin strategy. B) Subcellular localization of a second copy expressed under the control of the tubulin promoter. The proteins are detected using anti-Ty antibodies, whereas GAP45 staining shows the periphery of the parasites. Scale bar: 2 µm. See also Figure S3 for colocalization with Golgi, IMC, rhoptry and nucleus markers.

To assess expression of putative apicomplexan DHHCs, epitope tags were introduced at the C-terminus of the DHHCs at the endogenous loci (Figures 2 and S2A). In *T. gondii*, a triple Ty-tag was inserted by single homologous recombination in the 16 genes expressed in tachyzoite stage using the *KU80*-knockout (KO) strain 39,40. Typically, the level of expression detected was quite low, which might in part be explained by the difficulty in extracting and running on SDS–PAGE these polytopic proteins. Four of them (TgDHHC4, TgDHHC5, TgDHHC12 and TgDHHC17) were not detectable by immunoblot, whereas PCRs performed on genomic DNA confirmed both integration and clonality of the corresponding knockin strains (Figure S2B) and TgDHHC17 was weakly detectable by immunofluorescence (Figure 3A). Some of the proteins migrated aberrantly with respect to their predicted molecular weight and some showed more than one band (Figure 2A).

To tag *P. berghei* DHHCs C-terminally with a triple HA (3xHA) epitope tag, we used vectors from the *Plasmo*GEM resource (http://plasmogem.sanger.ac.uk) 41. Tagging vectors were available for 9 of the 11 PbDHHCs (PbDHHC3–11), all of which integrated successfully as demonstrated by Southern blotting of chromosomes separated by pulsed field gel electrophoresis (PFGE) (data shown for PbDHHC3-11, Figure S2D). Protein expression was detectable by immunoblotting of *P. berghei* schizont preparations for five of nine 3xHA-tagged DHHCs, PbDHHC3, 5, 7, 8 and 9 (Figure 2A). PbDHHC4, 6, 10 and 11 are either expressed at a level too low to be detected by tagging at the endogenous locus or have stage-specific profiles and are not expressed in *P. berghei* schizonts, which was the only parasite life cycle stage investigated in this study at present. Stage-specific expression is certainly a possibility in the case of PbDHHC6 and 10, where expression of the *P. falciparum* homologues appears to be more upregulated in gametocytes in RNASeq data 29.

### Subcellular localization of *T. gondii* and *P. berghei* DHHCs

Most of the endogenous DHHCs gave a signal by immunofluorescence assay (IFA) in *T. gondii* (Figure 3A). For the TgDHHCs for which no staining was detected, a second copy of the coding sequence under the control of the strong tubulin promoter was introduced (Figure 3B). As reported in human and yeast 42, several DHHCs are found to the Golgi apparatus (TgDHHC1, 5, 6, 9, 11, 12, 15 and 17). Golgi localization of TgDHHC1 was confirmed by co-staining with the Golgi protein GRASP 43 (Figure S3A). TgDHHC4 and TgDHHC13 are at the pellicle but not detectable in the IMC of the nascent daughter cells suggesting a localization at the PM. This subcellular compartment of the pellicle can be distinguished from the IMC by treatment of the parasites with the pore-forming *Aeromonas hydrophila* aerolysin 15–44. Upon separation of PM from IMC, TgDHHC13 staining colocalized with the surface GPI-anchored protein SAG1 and was distinct from the IMC (Figure S3B). TgDHHC3, 8 and 16 are localized to the ER (Figure 3). TgDHHC3 and TgDHHC8 appear as punctate staining around the nucleus suggestive of vesicles originating from the ER, whereas TgDHHC16 seems to be in the ER membrane around the nucleus as shown by its staining around the nuclear marker ENO2 45 (Figure S3A).

In addition to the compartment shared by other eukaryotic cells, three DHHCs were found in apicomplexan-specific organelles. TgDHHC7 is present at the rhoptry organelles, colocalizing with the rhoptry-bound, acylated protein TgARO 18 (Figure S3A). TgDHHC2 and TgDHHC14 are found to the IMC of the growing daughter cells. While TgDHHC2 is expressed in all the three subcompartments of the IMC 21, TgDHHC14 is excluded from the apical cap (Figures 3 and S3A) as shown by colocalization with GAP40, a polytopic protein of the IMC 15.

**Figure 4:**
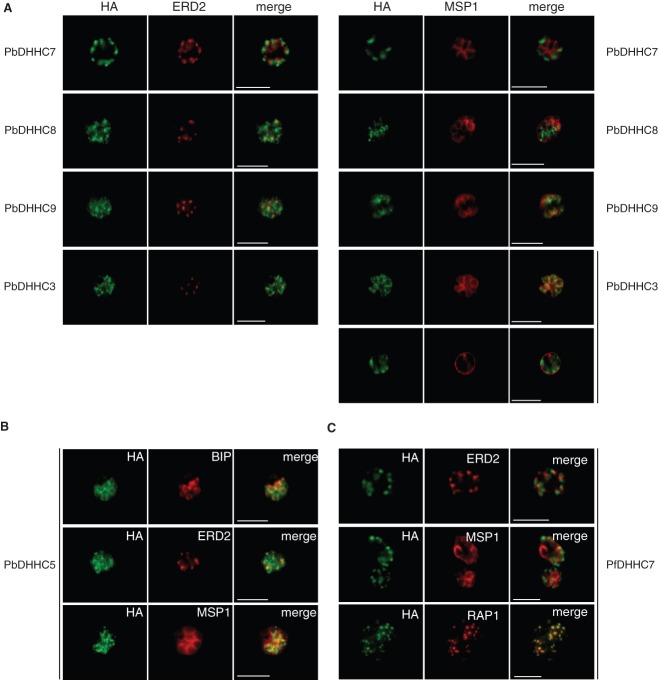
Localization of the DHHC-containing proteins in *Plasmodium.* A) Triple HA epitope-tagged *Pb*DHHC proteins were localized by immunofluorescence in relation to the Golgi marker ERD2 (left panel) and PM marker MSP1 (right panel). B) Triple HA epitope-tagged *Pb*DHHC5 was localized with the additional ER marker BiP. C) Detection of triple HA epitope-tagged *Pf*DHHC7 protein using an anti-HA antibody. *Pf*DHHC7 localizes to the rhoptry, as demonstrated by colocalizing with the PfRAP1 rhoptry marker. Scale bars: 5 µm.

PbDHHC3, 5, 7, 8 and 9 all could be localized to discrete foci in *P. berghei* schizonts (Figure 4A,B). PbDHHC8 did not colocalize with either ERD2 or MSP1 (Figure 4A), so its precise location is as yet unknown, and our ability to establish it is limited based on available markers. By contrast, PbDHHC3 and 9 appear to colocalize with MSP1 staining in late schizonts (Figure 4A), but this is not seen in earlier stages of schizogony (data shown for PbDHHC3, Figure 4A), suggesting possible IMC localization. PbDHHC5 does not colocalize with the Golgi marker, ERD2 46, or with the PM marker, MSP1 47, but partially colocalizes with BIP 48, suggesting an ER localization (Figure 4B). PbDHHC7 gave a punctate distribution, distinct from ERD2. Co-staining with MSP1 suggested that it was apically located and might be localized to the rhoptries like TgDHHC7 (Figure 4A). As no *P. berghei* rhoptry antibody was available to confirm the location, the endogenous copy of PfDHHC7 was tagged with a 3xHA in the *P. falciparum* 3D7 line. PfDHHC7 staining was also apical and colocalized with the rhoptry marker RAP1 49, confirming that the rhoptry location of this DHHC7 is conserved across apicomplexans (Figure 4C).

### A subset of the DHHCs are encoded by essential genes in *T. gondii* and *P. berghei*

To assess the importance of the DHHCs in *T. gondii* life cycle, the same knockin strategy as previously described for the C-terminal epitope tagging was used. However, in this case, the region of homology chosen for the recombination laid upstream the DHHC-CRD in order to create a truncated and hence non-functional protein (Figure S4A). Out of the 16 PATs expressed in tachyzoites, 11 were successfully disrupted as shown by the PCRs analysis on genomic DNA, thus confirming integration of the constructs and clonality of the strains (Figure S4B). Most of the truncations were not detectable by western blot or by IFA probably because they were unstable and degraded except for TgDHHC3 and TgDHHC8 for which the truncated proteins were detected at the expected sizes (Figure S4C) and by a punctate staining in the parasite (Figure S4D). The individual deletion of these 11 DHHC-containing proteins did not impact on the lytic cycle of the parasites as monitored by plaque assay (Figure S5A) or on their intracellular growth (Figure S5B). These DHHCs are localized to the Golgi, PM and ER/vesicles, where more than one DHHC was present, suggesting a possible functional redundancy. However, five genes coding for DHHCs could not be disrupted, although the loci were accessible to homologous recombination (introduction of a C-terminal epitope tag for localization by single crossing over), and therefore appeared to be critical for parasite survival. TgDHHC2 and TgDHHC14 are present at the IMC; however, they localized to distinct subcompartments of the IMC that likely reflect non-overlapping essential functions. TgDHHC7 is the only enzyme located to the rhoptries, whereas both TgDHHC5 and TgDHHC9 localized to the Golgi apparatus. However, in this latter case, the two proteins exhibit special features compared with the other members of the family present in the Golgi. TgDHHC5 shows a very pronounced cell cycle regulation of its mRNA 27 (Figure S1A), while TgDHHC9 possesses a signal peptide.

To establish which *Plasmodium* DHHCs are critical for the intraerythrocytic stages development, we used KO vectors from the *Plasmo*GEM resource (http://plasmogem.sanger.ac.uk) 41, which were available for PbDHHC3-11. After transfection, PFGE confirmed integration of the targeting vector into the expected chromosome in seven lines, PbDHHC3, 5–7 and 9–11 (Figure S2D). This indicates that these seven PbDHHCs, including four of the six genes for which no blood-stage expression was detectable by epitope tagging, are either functionally redundant for *P. berghei* asexual blood-stage growth or have a primary function in another life stage. Although KO vectors were available for PbDHHC4 and 8, following transfection no transgenic lines were obtained for these constructs. These data may indicate that PbDHHC4 and 8 could be essential for *P. berghei* blood-stage growth, although this would need to be confirmed by attempts to disrupt the locus whilst providing an episomally expressed copy of the gene.

### TgDHHC7 is essential for rhoptry organelle positioning and parasite invasion

**Figure 5:**
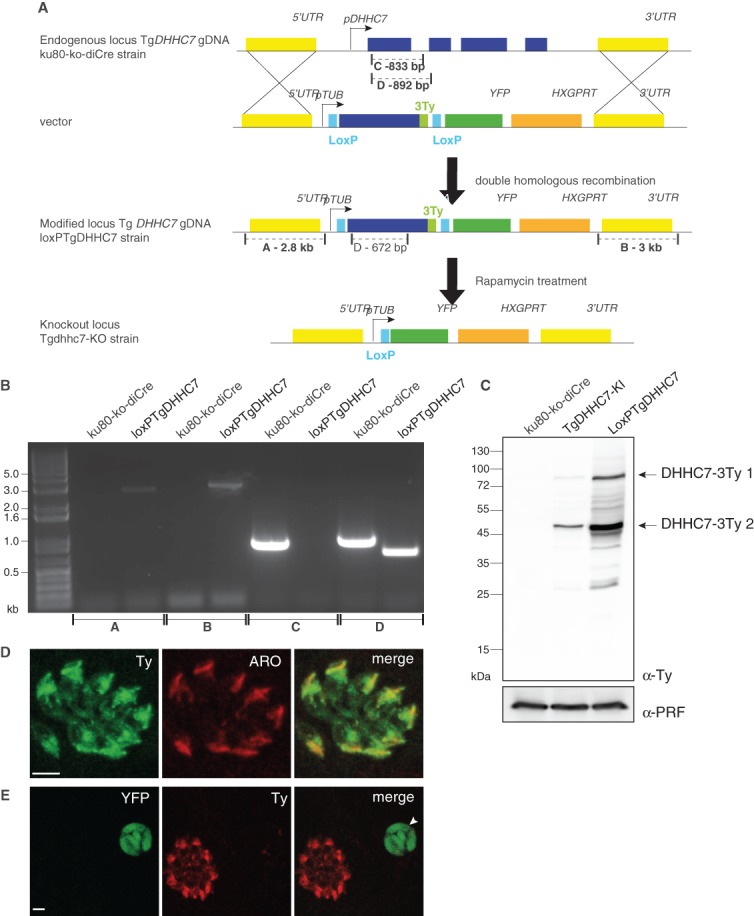
Generation of *TgDHHC7*-KO. A) Scheme of the diCre-LoxP strategy used to delete the *TgDHHC7* gene. B) Genomic PCR analysis of the ku80-ko-diCre and LoxPTgDHHC7 strains. The position of the primers and the expected sizes are shown on the scheme. C) Western blot performed with anti-Ty antibodies on total extracts of ku80-ko-diCre, TgDHHC7-KI and LoxPTgDHHC7 strains. Profilin (PRF) was used as a loading control, while TgDHHC7-3Ty 1 and 2 indicate two forms of TgDHHC7. D) Immunostaining of intracellular LoxPTgDHHC7-3Ty parasites with anti-Ty and anti-ARO antibodies. Scale bar: 2 µm. E) Immunostaining with anti-Ty antibodies of intracellular LoxPTgDHHC7-3Ty parasites 144 h after a 4-h rapamycin treatment. The arrowhead shows an excised vacuole not expressing TgDHHC7-3Ty compared with the other vacuole. Scale bar: 2 µm.

**Figure 6:**
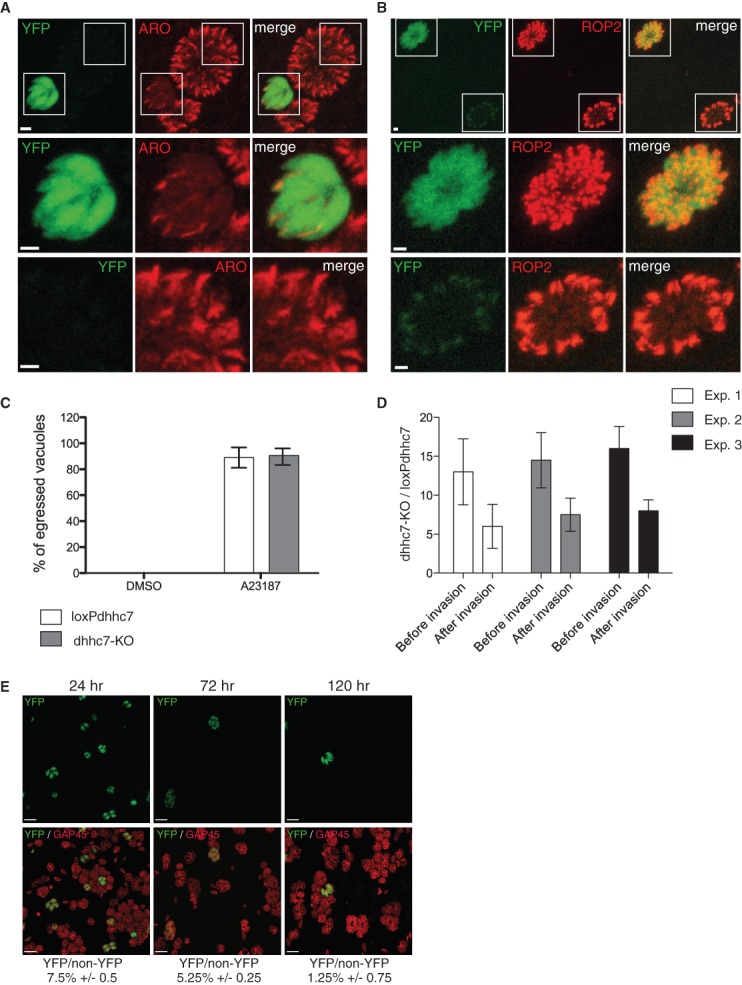
Deletion of *TgDHHC7* impairs rhoptry positioning and invasion. Immunofluorescence assay performed on intracellular LoxPTgDHHC7-3Ty parasites 144 h after a 4-h rapamycin treatment with anti-ARO (A) and anti-ROP2/4 antibodies (B). The middle and lower panels show a magnification of the upper ones according to the boxes. The middle panels show a magnification of *dhhc7*-ko vacuoles, whereas the lower ones show a magnification of a LoxPTgDHHC7-3Ty vacuole. Scale bar: 2 µm. C) Induced egress assay performed on loxPTgDHHC7 parasites 80 h after a 4-h rapamycin treatment. D) Invasion assay performed on loxP-TgDHHC7 parasites 50 h after a 4-h rapamycin treatment. E) Immunofluorescence assay performed on intracellular LoxPTgDHHC7-3Ty parasites every 48 h post-rapamycin treatment starting and showing a fast decrease of the excised parasites with the time (ratio YFP/non-YFP). Scale bar: 10 µm.

To investigate the function of TgDHHC7, a conditional deletion of the gene using a recently established strategy based on inducible Cre recombinase activation was applied 50. First, the *TgDHHC7* locus was replaced by double homologous recombination with a *TgDHHC7* cDNA expressing cassette flanked by two loxP sites (loxPTgDHHC7-3Ty) and under the control of the tubulin promoter in the ku80-ko-diCre strain (Figure 5A). The correct integration was checked by PCR on a clone (Figure 5B). LoxPTgDHHC7-3Ty localized to the rhoptries even if the expression level was higher than the endogenous one (Figure 5C,D). Upon addition of rapamycin, the excision of *TgDHHC7* was detectable by loss of signal with anti-Ty antibodies and the concomitant expression of the YFP-cassette in 10–15% of the parasites. After three passages (≈140 h), the YFP-expressing parasites were not detectable anymore by fluorescence microscopy. Importantly, TgARO, an armadillo repeat-containing protein recently shown to be anchored by palmitoylation in the membrane of the rhoptries and facing the cytosol, was a potential substrate for TgDHHC7 18. We have generated specific antibodies against TgARO 51 and found here that it becomes mainly cytosolic in Tgdhhc7-ko parasites (Figure 6A). This constitutes indirect evidence that TgDHHC7 acts as PAT for TgARO. In the absence of TgDHHC7, the rhoptries, stained with anti-ROP2/4 antibodies, were not found at the apical pole but dispersed throughout the parasite cytosol (Figure 6B). This phenotype recapitulates the inducible knockdown of TgARO 51. The Tgdhhc7-ko parasites could not be cloned and therefore the pool generated after excision had to be used to monitor the phenotypic consequences of TgDHHC7 deletion in egress and invasion by comparing the Tgdhhc7-ko (YFP-positive) with the loxPTgDHHC7-3Ty (YFP-negative). Induced egress in presence of the calcium ionophore A23187 was not affected in Tgdhhc7-ko parasites (Figure 6C) but in contrast the invasion dropped down to 49 ± 3% when monitored 48 h after rapamycin treatment (Figure 6D). At later time points after excision, the parasites are rapidly lost, which hampers a quantitative assessment of the phenotype upon depletion in TgDHHC7-Ty. Nevertheless, we have performed a longer term analysis on parasites every 48 h post-excision until 120 h and showed that almost no parasites lacking TgDHHC7 managed to invade host cells (Figure 6E).

## Discussion

All DHHC members are multipass TMD proteins and some contain ankyrin repeats, with the DHHC motif typically located between two transmembrane domains and facing the cytosol. The repertoire of DHHC-containing proteins in *T. gondii* and *Plasmodium spp*. is significantly larger compared with *S. cerevisiae*, which has only seven members reflecting the more elaborated endomembrane system in Apicomplexa and their specialization as professional secretory cells. Another protozoan parasite, *Trypanosome brucei*, possesses 12 DHHCs; however, none of them appeared to be essential based on RNA interference knockdowns 52. This suggests a functional redundancy for the palmitoylation of essential proteins although information on the localizations of these DHHCs and clean gene deletion is lacking to support such conclusion.

A comprehensive study of DHHC PAT location and essentiality has been carried out in yeast, but the only other example in eukaryotic cells used ectopic expression using non-endogenous promoters. In this study, we epitope tagged the endogenous genes for all 18 *T. gondii* DHHCs and all 11 *P. berghei* DHHCs. Expression of 16 of 18 DHHCs was detectable in *T. gondii* tachyzoites, while 5 of 11 DHHCs were detectable in *P. berghei* schizonts. The smaller number of *P. berghei* expressed DHHCs may reflect the more complex life cycle of this parasite, and the remaining DHHCs may be expressed in sexual and/or liver stages, where the parasite faces quite different environments and therefore different protein organization challenges.

**Figure 7:**
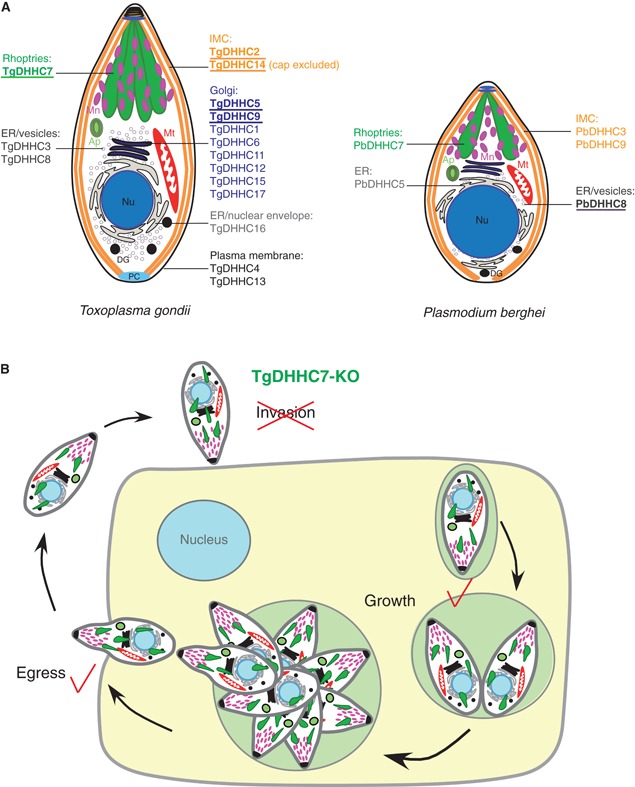
Summary of PATs localization and essentiality in *T. gondii* and *P. berghei.* A) Scheme of *T. gondii* tachyzoite (left panel) and *P. berghei* schizont (right panel) highlighting the location of the PAT in the different compartments of the respective parasites. The PATs identified as critical for the parasites are underlined. Ap, apicoplast; DG, dense granule; Mn, micronemes; Mt, mitochondrion; Nu, nucleus; PC, posterior complex. B) Schematic representation of the impact of TgDHHC7 deletion on *T. gondii* life cycle.

C-terminal epitope tagging of the endogenous locus revealed that DHHC proteins were distributed into at least six clearly distinct compartments. This included core elements of the secretory pathway, just as was found in *S. cerevisiae*, but more interestingly also included rhoptries and IMC, organelles that are unique to the phylum and play a key role in pathogenesis (Figure 7A). Just as with other aspects of the eukaryotic secretory pathway, apicomplexans have subverted elements of the palmitoylation machinery to regulate their invasive life cycle.

The profiles of expression of the DHHCs during the cell cycle are largely consistent with the timing of biogenesis of the organelles in which they are located and with the expression of other proteins resident within those organelles 27 (Figure S1). The one exception was TgDHHC5, which shows a totally distinct profile compared with the other Golgi DHHCs. TgDHHC5 transcript peaks at the time of cytokinesis when very few genes are transcribed. A gene coding for a protein phosphatase, homologous to TgGAP50 and which distributes between the Golgi and the apicoplast, shows the same expression profile as TgDHHC5. This single transmembrane domain protein has a strongly predicted palmitoylation site, and is a potential target for TgDHHC5 (J. Salamun, unpublished data).

The essential versus dispensable nature of each DHHC family member was then assessed by gene disruption attempts by single crossing-over upstream of the DHHC motif. Eleven of 16 tachyzoite-expressed *T. gondii* DHHCs were proven to be dispensable without significant impact on parasite fitness, and there was also evidence for redundancy in the case of 8 of 10 *P. berghei* DHHCs tested. Interestingly, three of the essential TgDHHCs are targeted to compartments found uniquely in Apicomplexa. The two IMC-specific DHHCs are essential, but given the fact that their localization is restricted to distinct subcompartments of the IMC, they probably act on different substrates. Potential substrates are IMCs 23 and ISPs 21,22 that are selectively anchored in these subcompartments. It is plausible that the substrate specificity of the PATs is the key determinant for the specific targeting of the IMCs and ISPs. However, the mechanism by which TgDHHC2 and TgDHHC14 end up in two subcompartments of the IMC is less clear and would involve a better understanding of the biogenesis of the IMC. No essential DHHC was identified at the *T. gondii* PM, which is somewhat surprising given that TgGAP45, which plays a crucial role in motility and invasion 15, is N-terminally palmitoylated at the PM. As both TgDHHC4 and TgDHHC13 that are present at the PM are dispensable, it is possible that they fulfil an overlapping function.

While in general there was little clear overlap in location and essentiality of DHHC homologues between *T. gondii* and *P. berghei*, DHHC7 found in the invasive rhoptry organelles in both *T. gondii* tachyzoites was also apically located in *P. berghei*, and was also confirmed to be in the rhoptries in the major human pathogen *P. falciparum*. As the DHHC-containing proteins are putative enzymes that are typically expressed at low level and in a cell cycle-dependent fashion, we opted for the use of the diCre-LoxP system to knock out the *TgDHHC7* gene by excision 50. The Cre-dependent deletion of TgDHHC7 is conveniently detectable by the concomitant repositioning of the YFP cassette in front of an active promoter. Despite several attempts to purify the Tgdhhc7-ko by fluorescence activate cells sorting and cloning by limiting dilution, we failed to isolate clone lacking the gene, indicating that this gene is critical for the propagation of the parasites (Figure 7B). The palmitoylated rhoptry protein TgARO has recently been identified as a key protein in the biogenesis/positioning of the rhoptries 51–53. Importantly, excision of *TgDHHC7* recapitulates the phenotype suggesting that this PAT has a dedicated role and possibly a very restricted range of substrates. To date, no other palmitoylated proteins have been reported at the surface of the rhoptry organelle. So far the failure in cloning Tgdhhc7-ko parasite had hampered further characterization of TgARO but clearly indicates that this PAT is needed for parasite propagation. Most recently, the conditional knockdown of *TgDHHC7* based on the tet-inducible system 54 has been reported 53. The results of this study are in accordance with the data presented here and unequivocally establish the importance of TgDHHC7 in invasion and identify TgARO as a substrate for TgDHHC7 53. While the two reverse genetic strategies led to the same conclusion, the modest yield of diCre excision limits the phenotypic investigations compared with the tet-system. However, the inability of propagating parasites lacking *TgDHCC7* gene upon excision provides a more definitive proof of the essential nature of the gene. Moreover, another study focusing on the functional dissection of TgARO formally establishes the importance of palmitoylation for TgARO function in positioning the rhoptry organelles to the apical pole of the parasite 51.

Interestingly, a KO line was successfully generated for *PbDHHC7*, with the caveat that dilution cloning of this line has not yet been performed. Initial experiments suggest that this line may have a slow growth phenotype, although confirmation of this will await clonal dilution. However, the fact that a *PbDHHC7* KO could be generated raises the question whether in *P. berghei*, more than one DHHC-PAT could perhaps be localized to the rhoptries and perform an overlapping function or if a Golgi-located PAT could compensate. Clearly, further investigation into this line will be required.

Apicomplexan parasites have clearly adapted the palmitoyl acyl transferase protein family for their own ends, just as they have many other aspects of the eukaryotic secretory pathway. Some elements are recognizable from model organism studies, with multiple DHHCs localized to the Golgi and early secretory pathway. However, other DHHCs are located to phylum-specific organelles where they are likely to play a specific role in host cell invasion. DHHC7 is highly conserved across the phylum of Apicomplexa suggesting that its role in rhoptry organelle biogenesis and consequently the critical implication for host cell invasion is a preserved mechanism. Given the importance of invasion for parasite survival and pathogenesis, this and other invasion-associated DHHCs clearly become interesting targets for inhibitor development.

## Materials and Methods

### Preparation of *T. gondii* genomic DNA and total RNA

Genomic DNA was prepared from tachyzoites (RH and ΔKU80 strains) using the Wizard SV genomic DNA purification system (Promega). RNA was isolated from tachyzoites using Trizol (Invitrogen). cDNA was then generated by reverse transcription-PCR performed with the Superscript II reverse transcriptase (Invitrogen) according to the manufacturer’s instructions.

### Annotation of the *T. gondii* DHHCs

All amplifications were performed with La Taq, Ex Taq (TaKaRa) or Phusion (NEB) DNA polymerases. The DHHC genes for which no EST or mass spectrometry data were available in the genome database (ToxoDB) to determine their full-length sequence were experimentally annotated from tachyzoite cDNA using the primers listed in Table S1. For each annotation, at least two clones have been sequenced.

### Amino acid sequence alignments and molecular phylogeny analyses

Sequences used in this study have all been obtained from the EuPathDB database 36. Multiple alignments of DHHC amino acid sequences were computed using muscle program 55. Only sequences conserved in all DHHC genes were included in the analysis (e.g. DPG, DHHC and NxTTxE regions of the DHHC protein sequences). The two alignments used for the phylogenetic analysis are presented in Figure S6 for Apicomplexan DHHCs and Figure S7 for *P. falciparum*, *P. berghei*, *T. gondii* and *N. caninum* sequence alignments. Phylogenetic analysis was performed by two methods, NJ 34 using the ‘number of differences’ model with pairwise removal of gap-containing sites (1000 bootstrap replicates were performed) and the second method by ML 56 using PhyML 57 with approximate likelihood-ratio test Shimodaira-Hasegawa-like (SH-like) 56 and variable time score matrix (VT) or Le and Gascuel (LG) 58 selected as amino acid substitution model as recommended by Prottest 3.2 59 analysis under Akaike Information Criterion framework (AIC) and Second Order Akaike framework (AICc). All the ML analyses have been performed on DIVEIN 60 and phylogenetic trees were visualized with Mega5 graphic tool 61. Bootstrap values obtained from these two different phylogeny analyses are indicated on the trees presented in Figures 1 and S1; only values above 95 were considered as significant allowing to form clusters represented by coloured boxes.

### Generation of *T. gondii* vectors

All amplifications were performed with Ex Taq (TaKaRa) or Phusion (NEB) DNA polymerases. For the knockin constructs (full-length and truncation upstream of the DHHC domain), around 1–1.5 kb genomic fragments of all TgDHHC genes except TgDHHC10 and TgDHHC18 were amplified by PCR using the primers listed in Table S2, and then digested with *Kpn*I or *Apa*I and *Nsi*I or *Sbf*I restriction enzymes and cloned into *Kpn*I or *Apa*I and *Nsi*I sites of the pTUB8MIC13-3Ty-HX vector 62. Before transfection, all the plasmids were linearized in the middle of the genomic fragments.

To express a second copy of TgDHHC3, TgDHHC5, TgDHHC6, TgDHHC12 and TgDHHC16, the corresponding coding sequences were amplified from cDNA with primers listed in Table S2 and cloned into pTUB8MycGFPPfMyoAtailTy-HX 63 between the *Eco*RI and *Nsi*I or *Sbf*I sites. The coding sequence of DHHC4 was cloned in three pieces in the same vector using primers TgDHHC4-F11/TgDHHC4-R15 for the N-terminal, TgDHHC4-F14/TgDHHC4-R12 for the middle part and TgDHHC4-F6/TgDHHC4-R8 for the C-terminal part.

To generate the 5′DHHC7-Tub8-loxP-TgDHHC7-3Ty-loxP-YFP-3′DHHC7 vector, around 2–2.5 kb genomic fragments of 5′ and 3′flanking regions of TgDHHC7 were amplified from genomic DNA using primer pairs TgDHHC7-F38/TgDHHC7-R33 and TgDHHC7-F34/TgDHHC7-R35, respectively. The 5′ fragment was cloned into *Kpn*I/*Apa*I sites of the Tub8-loxP-KillerRed-loxP-YFP 50 and the 3′ fragment into the *Sac*I site. The cDNA of TgDHHC7 has been amplified with primers TgDHHC7-F13 and TgDHHC7-R5, cloned into pTUB8MIC13-3Ty-HX 62 between *EcoR*I and *Nsi*I sites and then subcloned into 5′DHHC7-Tub8-loxP-KillerRed-3Ty-loxP-YFP-3′DHHC7 between *EcoR*I and *Pac*I restriction sites.

### *T. gondii* culture, parasite transfection and selection of stable transformants

*Toxoplasma gondii* tachyzoites [RH*hxgprt*^−^, ΔKU80*hxgprt*^−^ 39,40 and diCre-ΔKU80*hxgprt*^−^ strains 50] were grown in confluent human foreskin fibroblasts (HFFs) maintained in DMEM (Invitrogen) supplemented with 5% foetal calf serum, 2 mM glutamine and 25 µg/mL gentamicin. Parasite transfections were performed by electroporation as previously described 64. The hypoxanthine-xanthine-guanine phosphoribosyl transferase (*hxgprt*) gene was used as a positive selectable marker in the presence of mycophenolic acid (25 mg/mL) and xanthine (50 mg/mL) as described before 65.

### Production of PbDHHC vectors

*Plasmodium berghei* gene targeting vectors were obtained from the open access *Plasmo*GEM resource hosted at the Wellcome Trust Sanger Institute (http://plasmogem.sanger.ac.uk/). *Plasmo*GEM vectors are constructed as previously described 41. Targeting cassettes were released from the backbone of the linear N15 phage pJAZZ vector 66 by digestion with NotI and 2 µg was transfected by electroporation of purified schizonts as described 67. Unique identification numbers for all *Plasmo*GEM vectors are shown in Table S3. All vector and primer sequences used are available at http://plasmogem.sanger.ac.uk/.

### Generation and genotyping of PbDHHC transgenic lines

The KO or 3xHA epitope tagging constructs were transfected into *P. berghei* 2.34 ANKA purified schizonts, with transfectant parasite populations injected intraperitoneally into Theiler’s Original mice, and transgenic lines selected for by administration of pyrimethamine in drinking water 67,68. Stable parasitaemia of pyrimethamine-resistant parasite lines was typically detected on day 7–8 post-transfection. After a second round of drug selection, parasites were isolated from the blood of infected mice and genomic DNA was obtained using standard methods. The genomic DNA preparations were subjected to PCR to confirm the presence of the correct targeting cassette, using the generic primer GW2 in combination with the gene-specific primer QCR2 (data not shown). Primer sequences are all available at the *Plasmo*GEM website associated with the vector design as listed in Table S3. Southern hybridization of chromosomes separated by PFGE was used to confirm that integration had taken place. As a probe we used a 500-bp PCR-amplified fragment recognizing the 3′UTR of the *Pbdhfr-ts* gene, which is present twice in each targeting or tagging vector and once at the endogenous *dhfr-ts* locus on chromosome 7.

### Generation of the PfDHHC7 3xHA line

A 1132-bp fragment of the 3′ region of the PfDHHC7 open reading frame was PCR amplified (forward primer—GCCTGCAGGGAGAACGAAGACATTGTAAATGG and reverse primer –GCGCTCGAGTATATTTGTTTTTATTGGAATAATTTCC). The PCR fragment was then introduced into the pCAM-BSD-3HA vector. Transfection of ring-stage *P. falciparum* 3D7 parasites was performed as previously reported 69. Positive drug selection was started 1 day post-transfection with 2.5 µg/mL blasticidin-S and maintained until stable parasite growth was obtained. To select for parasites that had integrated the construct via homologous recombination, parasites were grown without drug pressure for 3 weeks, after which drug pressure was reapplied until stable parasite growth was once again established.

### Immunodetection of Ty-tagged *T. gondii* proteins

For IFA, parasites-infected HFF cells were fixed with 4% paraformaldehyde (PFA) or 4% PFA/0.05% glutaraldehyde (PFA/GA) in PBS, depending of the antigen to be labelled and processed as previously described 70. The primary antibodies used were mouse α-Ty (mAb BB2) 71, rabbit α-GAP45 72 and α-ARO 51. Confocal images were collected with a Leica laser scanning confocal microscope. Stacks of sections were projected using the maximum projection tool. For western blot analysis, pellets from extracellular tachyzoites were resuspended in RIPA buffer and incubated on ice for 15 min before centrifugation at 14 000× ***g*** for 15 min at 4°C. The supernatant was then mixed with loading buffer under reducing condition and resolved by SDS–PAGE. Ty-tagged proteins were detected using mouse α-Ty (mAb BB2) 71 primary antibody and anti-mouse HRP (Sigma) secondary antibody. Loading control was done using rabbit α-PRF 72 and anti-mouse HRP (Sigma) secondary antibodies.

### Immunodetection of HA-tagged *Plasmodium* proteins

*Plasmodium berghei* schizonts were prepared as described for transfections 67 and *P. falciparum* schizonts were obtained from *in vitro* cultures. Cells were fixed in 4% formaldehyde/0.01% GA/PBS for 15 min (*P. berghei*) or 60 min (*P. falciparum*), then permeabilized in 0.1% Triton X-100/PBS for 10 min and blocked in 3% BSA/PBS for 60 min prior to immunodetection using the following primary antibodies: α-HA (mouse or rabbit, Cell Signalling Technologies) at 1:200, α-ERD2 (rabbit) at 1:2000, α-MSP1 (mouse) at 1:2000 and α-RAP1 (mouse) at 1:1000. The secondary antibodies used were α-mouse or rabbit Alexa Fluor® 555 at 1:500 and α-mouse or rabbit Alexa Fluor® 488 at 1:1000. All antibodies were diluted in 1% BSA/PBS. Nuclear DNA was stained with 4′,6-diamidino-2-phenylindole (DAPI) and cells mounted in Prolong antifade mounting reagent (Life Technologies). For western blot analysis, purified schizont pellets were boiled in denaturing buffer for 5 min, and HA-tagged proteins were detected using rabbit α-HA (Cell Signalling Technologies) primary antibody at 1:400 and anti-rabbit HRP (Amersham) secondary antibody at 1:4000, both diluted in 2% FBS and PBS.

### *T. gondii* egress assay

Extracellular loxPTgDHHC7-3Ty-expressing parasites were treated with 50 mM of rapamycin for 4 h before their inoculation on host cells. Forty-eight hours later, freshly egressed parasites were allowed to grow for 30 h on new host cells. Egress was then stimulated for 8 min at 37°C with DMEM containing 3 μM of the Ca^2+^ ionophore A23187 from *Streptomyces chartreusensis* (Calbiochem) or 0.06% of dimethyl sulphoxide before fixation. IFA was performed using α-GAP45 antibodies. The average number of YFP and non-YFP egressed vacuoles was determined by counting 100 vacuoles in duplicate for three independent experiments.

### *T. gondii* invasion assay

Invasion assays were performed using the non-YFP strain as internal standard. Extracellular loxPTgDHHC7-3Ty-expressing parasites were treated with 50 mM of rapamycin for 4 h before their inoculation on host cells. Forty-eight hours later, freshly egressed parasites were passed on gelatin-coated coverslips and fix after 90 min to determine the ratio of YFP (*DHHC7*-KO)/non-YFP (loxPTgDHHC7) parasites. At the same time the parasites were transferred on new host cells and allowed to invade for 90 min at 37°C before washing. Then, incubation continued for 24 h and cells were fixed. Parasites were stained with α-GAP45 antibodies and the ratio between YFP and non-YFP parasite vacuoles was calculated. The efficiency of invasion was determined by counting at least 100 vacuoles in duplicate for three independent experiments.

## Notes

Figure S1 Evidence of the expression of 16 DHHCs by the tachyzoite stage of *T. gondii* and analysis of the conservation across the phylum Apicomplexa. A) Cell cycle profile based on the transcriptomics across the intracellular tachyzoite cell cycle (strain RH) provided on ToxoDB (1) by Behnke et al. (2). For comparison, ROP17 and GAP40, typical proteins of the rhoptries and IMC organelles, respectively, have been added. B) Phylogenetic tree of apicomplexans DHHC-containing proteins based on neighbour-joining (NJ) distance analysis on one hand and on maximum likelihood (ML) on the other hand. Only nodes supported by a bootstrap value >80 are indicated and values >95 were considered as significant allowing to cluster sequences (coloured boxes). Protein accession numbers are given according to the EuPathDB website (1). Sequence alignment used to compute the phylogenetic tree is presented in Figure S6.

Figure S2 Genotyping of TgDHHC triple Ty-tagged transgenic lines, *Pb*DHHC triple HA-tagged and *Pb*DHHC KO transgenic lines. A) Scheme of the strategy used for the C-terminal tagging of the endogenous copy of the TgDHHCs in the ?KU80 strain. B) Genomic PCR analysis confirming the integration of the construct (amplified fragment B) and the clonality of the strains (non-amplified fragment A) for which no signal was detected by western blot and/or immunofluorescence assay. C) Scheme of the strategy used for triple HA tagging and for generation of the KO cell lines showing the position of the primers used for analysis. D) Pulsed field gel electrophoresis (PFGE) and Southern blot analysis of size separated *P. berghei* chromosomes using a probe specific to the *Pbdhfr* 3’UTR, demonstrating integration of the respective targeting vectors into the expected chromosomes.

Figure S3 **Colocalization of some *T. gondii* DHHC-containing proteins with specific markers of organelles**. A) TgDHHC1 colocalizes with GRASP-YFP, a marker of the Golgi apparatus, TgDHHC7 colocalizes with the rhoptry staining of TgARO, TgDHHC14 colocalizes with TgGAP40 staining in the growing daughter cells and TgDHHC16 staining is around the nuclear staining of ENO2. B) Aerolysin-treated parasites. The staining of TgDHHC13 colocalizes with the plasma membrane marker SAG1 and not with the IMC marker GAP45. Scale bar: 2 μm.

Figure S4 Eleven DHHC-containing proteins can be individually disrupted in *T. gondii*. A) Scheme of the strategy used to disrupt the DHHC genes in the ?KU80 strain. The homologous recombination takes place upstream of the DHHC motif to create a truncated and non-functional version of the protein. B) Genomic PCR analysis (or cDNA PCR analysis for TgDHHC11) confirming the integration of the constructs and the clonality of the strains. C) Western blot analysis showing that the truncated proteins TgDHHC3 and TgDHHC8 can be expressed by the parasites. D) The vesicular staining of the two truncated proteins TgDHHC3 and TgDHHC8 in the parasite is the same as the full-length corresponding proteins. Scale bar: 2 μm.

Figure S5 **Eleven *T. gondii* DHHC-containing proteins are not critical for tachyzoite survival**. A) Plaque assay stained with GIEMSA 7?days after invasion of the host cells with ?KU80, KI-DHHCs and KO-DHHCs. Scale bar: 0.4?mm. B) Intracellular growth assay performed by counting the parasites 24?h after invasion of the host cells.

Figure S6 **Alignment of the conserved domains used for the complete phylogenetic analysis**.

Figure S7 **Alignment of the conserved domains used for the complete phylogenetic analysis including *T. gondii*, *N. caninum*, *P. falciparum* and *P. berghei***.

Table S1 Primers used in this study for annotation of TgDHHCs. F, forward; R, reverse

Table S2 Primers used in this study for cloning of TgDHHCs. F, forward; R, reverse; the restriction sites are underlined

Table S3 Summary of the PlasmoGEM data available for the PbDHHCs

Table S4 Primers used in this study to check integration of *T. gondii* constructs. F, forward; R, reverse. A and B are the position of the primers on the scheme of Figure S2 for the knockin strategy at the C-terminal par of the genes, of Figure S4 for the knockin strategy upstream of the DHHC motif and of Figure 5 for the knockout with the DiCre-lox system

Table S5 Primers used in this study to check integration of *P. berghei* constructs. The position of the primers is shown on the scheme of Figure S3

Appendix S1 Supplemental experimental procedures
