# Supplementary material for: Global Analysis of Apicomplexan Protein S-Acyl Transferases Reveals an Enzyme Essential for Invasion
Source: Traffic. 2013 May 29;14(8):895–911. doi: 10.1111/tra.12081 (PMC3813974; doi:10.1111/tra.12081)
Supplement: Figure S3 — Colocalization of some T. gondii DHHC-containing proteins with specific markers of organelles. A) TgDHHC1 colocalizes with GRASP-YFP, a marker of the Golgi apparatus, TgDHHC7 colocalizes with the rhoptry staining of TgARO, TgDHHC14 colocalizes with TgGAP40 staining in the growing daughter cells and TgDHHC16 staining is around the nuclear staining of ENO2. B) Aerolysin-treated parasites. The staining of TgDHHC13 colocalizes with the plasma membrane marker SAG1 and not with the IMC marker GAP45. Scale bar: 2 μm. [file tra0014-0895-sd3.doc]

**Table S3.** Summary of the *Plasmo*GEM data available for the PbDHHCs

| PbDHHC | Pb gene ID | Design | | PlasmoGEM Design ID | PlasmoGEM  Vector Available | PbG clone ID |
| --- | --- | --- | --- | --- | --- | --- |
| PbDHHC3 | PBANKA_092730 | TAG | PbGEM-094114 | | YES | PbG02_B-22c03 |
| PbDHHC3 | PBANKA_092730 | KO | PbGEM-111866 | | YES | PbG02_B-22c03 |
| PbDHHC4 | PBANKA_142090 | TAG | PbGEM-065194 | | YES | PbG01-2325c05 |
| PbDHHC4 | PBANKA_142090 | KO | PbGEM-065186 | | YES | PbG01-2325c05 |
| PbDHHC5 | PBANKA_133780 | TAG | PbGEM-058319 | | YES | PbG01-2389d06 |
| PbDHHC5 | PBANKA_133780 | KO | PbGEM-072266 | | YES | PbG01-2389d06 |
| PbDHHC6 | PBANKA_083330 | TAG | PbGEM-112088 | | YES | PbG02_A-48e04 |
| PbDHHC6 | PBANKA_083330 | KO | PbGEM-027807 | | YES | PbG01-2385g10 |
| PbDHHC7 | PBANKA_124300 | TAG | PbGEM-052430 | | YES | PbG01-2474f11 |
| PbDHHC7 | PBANKA_124300 | KO | PbGEM-104070 | | YES | PbG02_A-56f05 |
| PbDHHC8 | PBANKA_141970 | TAG | PbGEM-065002 | | YES | PbG01-2428e06 |
| PbDHHC8 | PBANKA_141970 | KO | PbGEM-225987 | | YES | PbG01-2428e06 |
| PbDHHC9 | PBANKA_093210 | TAG | PbGEM-121234 | | YES | PbG01-2467c05 |
| PbDHHC9 | PBANKA_093210 | KO | PbGEM-121226 | | YES | PbG01-2467c05 |
| PbDHHC10 | PBANKA_051200 | TAG | PbGEM-112097 | | YES | PbG02_A-56e08 |
| PbDHHC10 | PBANKA_051200 | KO | PbGEM-015165 | | YES | PbG01-2347f08 |
| PbDHHC11 | PBANKA_031260 | TAG | PbGEM-225995 | | YES | PbG01-2356c07 |
| PbDHHC11 | PBANKA_031260 | KO | PbGEM-121242 | | YES | PbG01-2356c07 |
