# Supplementary material for: Global Analysis of Apicomplexan Protein S-Acyl Transferases Reveals an Enzyme Essential for Invasion
Source: Traffic. 2013 May 29;14(8):895–911. doi: 10.1111/tra.12081 (PMC3813974; doi:10.1111/tra.12081)
Supplement: Figure S5 — Eleven T. gondii DHHC-containing proteins are not critical for tachyzoite survival. A) Plaque assay stained with GIEMSA 7?days after invasion of the host cells with ?KU80, KI-DHHCs and KO-DHHCs. Scale bar: 0.4?mm. B) Intracellular growth assay performed by counting the parasites 24?h after invasion of the host cells. [file tra0014-0895-sd5.doc]

**Table S5. Primers used in this study to check integration of *P. berghei* constructs.**

All primers used to confirm the presence of the *Plasmo*GEM targeting vector following transfection are listed on the *Plasmo*GEM website, plasmogem.sanger.ac.uk, associated with the specific vector design. *Plasmo*GEM design IDs are listed in Table S3, and the position of the primers is shown on the scheme of figure S2C.
