# Supplementary material for: Global Analysis of Apicomplexan Protein S-Acyl Transferases Reveals an Enzyme Essential for Invasion
Source: Traffic. 2013 May 29;14(8):895–911. doi: 10.1111/tra.12081 (PMC3813974; doi:10.1111/tra.12081)
Supplement: Figure S6 — Alignment of the conserved domains used for the complete phylogenetic analysis. [file tra0014-0895-sd6.pdf]

```
                          10        20        30        40        50        60        70
                  ....|....|....|....|....|....|....|....|....|....|....|....|....|....|....|....
PBANKA_010830     CASTDPGKVP-YCKICNVWK-PDRTHHCSACNRCVLNMDHHCPWINNCVGFYNRRFFMQLLFY-HLKLISKNSTTIEN
PBANKA_031260     CLLKNPGYLN-YCKKCNFSK-IKRAHHCSVCNKCVKKMDHHCTWINNCVGLYNQKFFILLNIY-QYWGIKTNTSGIEF
PBANKA_040200     LYFKNPGFYK-VCVTCNIIK-PPRVHHCADCFHCVVHQDHHCVWVDNCIGINNQRSFYMFILS-NTKTILTNVTFYEH
PBANKA_051200     TYNISPGYVP-YCAYSKIYK-PDRSHYCRAIDKTVLKMDHYCPWVANCIGFYNYKFFLLSLLY-HLYLTSQNYTTLEF
PBANKA_083330     SSLCNPGYIT-YCIQCDLVQ-MLRSKHCKYCKQCIKTYDHHCLWINNCVGENNRLIFFLYLYF-HTHLCMVNETTWEN
PBANKA_092730     VTSINPIDPL-ECDICGFVE-PQ-SKHCKVCNKCVSVFDHHCMWVNNCIGKKNYKYFVSLLLA-HIYLISKKMTTYEY
PBANKA_093210     CSACNPGYVD-ICDKCNYLVRPERAHHCRTCQRCVLKMDHHCPWIGTCVGEKNLKFFFLFLIY-YVYFISHNITTIES
PBANKA_124300     SIVTPPGFIP-FCKWCCKYK-PDRAHHCRICKTCILKMDHHCPWIYNCIGYNNHKYFMLSLIY-HLWLTFKNMTTIEF
PBANKA_133780     VVSSNPGFLD-LCPTCFLFK-NTRTKHCAFCDKCIDIFDHHCIFTLNCMGIDNARIFLSWILS-SIFNILENITLNEK
PBANKA_141970     TAFCDPGIIP-WCVHCNHFK-EPRSKHCYTCNNCVTKFDHHCVWLGNCIGIRNYRNFIFFILN-HLKIILLNKTTYED
PBANKA_142090     TSFMDPGIIP-YCYTCNIYR-GIRTVHCSICDNCVEKFDHHCPWVGNCIGARNYKYFIYFIFN-HIYTIVTNQTTYEQ
PF3D7_0932500     VSYRNPGYVT-YCKICDVYQ-ILRSKHCQMCKRCVRTFDHHCPWINNCVAENNRSFFLLYLYF-HSYLCLINETTWEN
PF3D7_1027900     TYKVNPGNIP-YCIHEKKYK-PDRSHYCRAIEKNVLKMDHYCPWVANCVGFYNYKFFLLSLFY-HIYLTSKNYTTLEF
PF3D7_1321400     TAFCDPGIIP-WCVNCNHFK-EPRSKHCYTCNNCVTKFDHHCVWIGNCVGNRNYRRFFFFILN-HLQMVLQNKTTYED
PF3D7_1322500     VVTSNPGYLE-LCPTCFLFK-NIRTKHCSLCDKCVEIFDHHCDFTLNCMGIENARIFLLWILL-SIMNILENITSNEK
PF3D7_0202900     CSLSDPGKIS-KCKTCNIIK-PARSKHCSYCSSCISRYDHHCFLLNNCIGGYNNMYYLVFLHI-IYFSLFNNITQNEL
PF3D7_0215900     CLCTNPGFLN-MCKKCNLLK-IKRSHHCSVCDKCIMKMDHHCFWINSCVGLYNQKYFILLNFY-QYCAIKTNTTGIEL
PF3D7_0303400     LYFKSPGFYK-ICVTCNIIK-PPRVHHCAECFHCIVHQDHHCVWVDNCIGIKNQRCFYMFIFC-NTRTILTNVTFYEH
PF3D7_0528400     SIIVSPGSIP-HCKWCCKYK-PDRTHHCRVCKSCILKMDHHCPWIYNCVGYNNHKYFMLSLIY-HIWLLINAMTTIEF
PF3D7_0609800     CASTDPGKVP-YCKICNVWK-PDRTHHCSACNRCVLNMDHHCPWINNCVGFFNRRFFIQLLFY-HLKLISKNSTTIEN
PF3D7_0714300     TSFMDPGIIP-YCYTCNIYR-GIRTVHCSICDNCVEKFDHHCPWVGNCIGARNYKYFVYFVFN-HIYTIVTNQTTYEQ
PF3D7_1115900     CSIVNPGYVD-ICDKCDFLVRPERAHHCRTCNKCILKMDHHCPWIGTCVGEKNLKFFFLFLIY-YIYFISKNITAIES
PF3D7_1121000     VTKTNPVDPL-QCDICGFVQ-PE-SKHCKVCNKCVSVFDHHCMWVNNCIGKKNYKYFVGLLST-HIFLISKKMTTYEY
TGME49_050870     AVFVSPGGVP-ECKWCLH---PDRTHHCRVCRTCVLKMDHHCPWIDNCVGWGNHKYFMLSIIY-HLYLMVKGMTTIEF
TGME49_078850     AVCTDPGRVP-YCKVCNV---PDRTHHCSACGRCVLNMDHHCPWINNCVGFYNRKYFIQLLIY-HLNLVLKNSTTIEN
TGME49_017870     CALRDPGEVS-TCTHCLR---PERAHHCSICNKCVMRMDHHCPWVGNCVGFNNYKQFLLFNFYTHLYYVLVNMTTIE-
TGME49_013550     TACSDPGILP-FCTTCNI---PPRSVHCAICDNCVERFDHHCPWLGNCIGLRNYRTFIFFVIF-HGYLIATNQTTYEQ
TGME49_024290     VAFGDPGIIP-WCTTCYF---PPRSKHCSVCNNCVRRFDHHCPWVSNCVGERNYRIFFFFLLL-NIYLIVNNRTTNEE
TGME49_024310     TAFSDPGIIP-WCTTCCL---PPRTKHCSTCDNCVQRFDHHCPWVSNCIGQRNYRVFFFFVFF-NFYLILNNLTTNED
TGME49_052200     AVVTPPGSIP-HCKWCRR---PDRAHHCRVCRQCVLKMDHHCPWIYNCVGWRNHKYFMLSLIY-HTHLVCNGMTTIEF
TGME49_055650     CSLKNPGIIN-ECSTCRF---PARSKHCRLCNVCVARFDHHCVWIGNCVGARNHGVFIIFLIT-HLYTAVWRNSTTNE
TGME49_069150     AVFTDPGSTK-QCSPCKGR--PPRAHHCKVCKECIFRMDHHCPWINNCVGLMNQKYFILFLIYSEQWEALETNTTLVE
TGME49_101370     ACVTGAGSVP-YCHKCAH---PDRAHHSRHTGTCTLKLDHYCPWVANDIGYFNYKYFYLTLLY-HTYLLSINSSTVEY
TGME49_084170     CLFSDPGAVP-SCRKCCS---PARAHHCSVCQRCILKMDHHCPWVNNCVGQTNQKFFLLFLVYFDQLSAIRANTTGIE
TGME49_029160     TVLKDPGIPR-FCRECRI---PAGSVHCDDCRVCIEGYDHHCPWTSKCVGKGNSKEFHAWIIL------------ED
TGME49_049380     ATTTDPIDPV-ECDVCG----HERSKHCRVCNKCVDGFDHHCMWINNCVGEKNYRPFFALLVF-HIYLVRHHLTTFEY
TGME49_093730     TYSGDAGIAP-VCVTCAI---TPRIHHCADCGHCLERQDHHCVWVDTCIARNNFQPFWFFLLC-TTRVMFTNVTYYEF
TGME49_093220     TALGDPGYLK-YCEICAM---PLRTKHCGHCGRCTRTHDHHCPWIGTCVAEENRVYFYWFLFL-HTYLMLSNLTTWES
TGME49_066940     ACTVDPGRPP-LCSTCGG---PLRTHHCRICNRCVLKQDHHCPWLNQCVGLHNYRFFFLFLFFFHVYLLMGNQTTIEV
TGME49_072320     LLLSDPGIHP-LCRTCWI---PLRTKHCSICDRCVDGFDHHCVWLYNCVGSLNARLFTAWLLLGHLRNIAQNITANEV
TGME49_046650     WTGINPGYIR-FCADCRI---PLRTRHCKECDHCVLTYDHHCAFLGCCVGEFNHWRFYLFLLS-HTYLMLSNQTTWVW
NCLIV_000160      TAVGDPGYLK-FCQICFM---PLRTKHCSQCGRCTRTHDHHCPWIGTCVAEENRVYFYWFLLL-HTYLMLSNLTTWES
NCLIV_000720      VVALMPGVAT-VCVTCAI---TPRIHHCADCGHCLERQDHHCVWVDTCIARNNVQPFWFFLLS-TTRVMFTNVTYYEY
NCLIV_039120      ACAVDPGRPP-HCVKCGA---PPRSHHCRICNRCVLKQDHHCPWLNQCVGLHNYRFFFLFVFFFHVYLLLGNQTTIEM
NCLIV_030100      TVLKDPGIPR-FCRDCQI---PPGSVHCDDCRVCIEGYDHHCPWTSKCVGKGNIWEFHVWIILIFYYGLTAAFLGQEE
NCLIV_063440      WVGINPGYIR-FCIYCRV---PLRTRHCAECNHCVLTYDHHCAFLGCCIGEFNHWRFYLFLLS-HTYLILSNQTTWD-
NCLIV_065580      ASTTDPIDPI-ECDVCG----NERSKHCRVCNKCVDGFDHHCMWINNCVGDKNYRPFFVLLVA-HIYLVLHHLTTFEY
NCLIV_034970      LLLSDPGTLP-LCRTCWI---ALRTKHCFVCNRCVEGFDHHCVWVYNCVGALNARLFTSWLLVGHMRNIAQNITANEV
NCLIV_037240      AIFTDPGSTK-HCSPCRGS--PPRAHHCKVCKECIFRMDHHCPWINNCVGLMNQKYFILFLIYSEQWEALETNTTLVE
NCLIV_048280      VAFSDPGIIP-WCTTCYL---PPRSKHCSVCNNCVRRFDHHCPWVSNCVGERNYRIFFFFLVF-NIYLIANNRTTNEE
NCLIV_062610      CALRDPGEVS-TCAHCLR---PERAHHCSICNKCVMRMDHHCPWVGNCVGFNNYKQFLLFNLYTHLYYVLVNMTTIE-
NCLIV_066400      AVFVSPGGVP-ECKWCMH---PDRTHHCRVCRTCVLKMDHHCPWIDNCVGWGNHKYFMLAVIY-HLYLVVKGMTTIEF
NCLIV_067160      AVCTDPGRVP-YCKVCNV---PDRTHHCSACGRCVLNMDHHCPWINNCVGFYNRKYFIQLLIY-HLNLVLKNSTTIEN
NCLIV_068450      VAFSDPGIIP-WCTTCYL---PPRSKHCSVCNNCVRRFDHHCPWVSNCVGERNYRIFFFFLVF-DIYLIANNRTTNEE
NCLIV_069490      TACSDPGILP-FCTTCNI---PPRSVHCAICDNCVERFDHHCPWLGNCIGLRNYRTFVFFVIF-HGYLISTNQTTYEQ
NCLIV_029260      CSLKDPGVID-KCSTCLF---PARSKHCRLCNVCVARFDHHCVWIGNCVGARNHGAFIVFLVT-HIYTAVWRNSTTNE
NCLIV_028970      CLLSDPGAVP-SCRKCRS---PARAHHCSVCQRCILKMDHHCPWINNCVGQTNQKFFLLFLVYFDQLSAIRSNTTGIE
NCLIV_007310      AVITPPGSIP-HCKWCRR---PDRAHHCRVCRQCVLKMDHHCPWIYNCVGWRNHKYFMLSLIY-HTHLVCNGMTTIEF
BBOV_III002420    VCASNPGFLE-YCVTCHIYR-PPRTVHCSSCGGCVLRYDHHCPYVANCIGFNNYRRFSYFVAT-HVVIIVKGQSTYDR
BBOV_III005080    CATVDPGRVP-ICKWCILYK-PDRAHHCTVCGRCVLMMDHHCPWVHNCIGWGNHKYFYLCLFY-HIWLMCEAYTTIEF
BBOV_II001280     TFASDPGKAR-VCITCNMIR-APRTHHCGSCGVCIVRQDHHCAWVDNCVGKGNQRSFCVFIAS-MVRNMTTDVTFYEF
BBOV_II001940     SSNTDPGSVP-YCKVCNVWK-PDRTHHCSACNRCVLNMDHHCPWISNCVGFYNRKCRDFASSW-HFRLVLKNSTTIEN
BBOV_II002930     VSYAEPGIIP-YCHTCNIYR-PPRSVHCSVCDVCVHKFDHHCKWLGNCIGGKNHKAFYGFLFF-HTYLICVNKTTNEQ
BBOV_IV003570     CTLSDPGYVP-DCPKCRSVR-PRRAHHCSVCKRCIIHMDHHCPWVGNCVGLFNQKFFIQYTVV-QLVNIYRNRTAIDK
BBOV_I002780      TAITDPGYIP-YCEYEDCYK-PDRAHYCRQLGRNVLKMDHYCHWMANCIGFYNYKFFFLTLFY-HLWYVADFDKQDDY
BBOV_I004680      SIHQDPGYRG-MCDIC-KSV-DASSKHCNICNKCVLRFDHHCIWVNNCIGAQNYKVFVALVAS-HCYLIHKKLTTYEY
TP01_0341         LNKSDPGVIP-WCQKCKIYR-PPRAKHCYICNRCIRRFDHHCYFLSNCIGHNNYKKFVFFIFL-NKYLILKNLTTYEY
```

```
TP01_0569       NTLKTPSRIF-YCRNENCYK-PDRAHYCRPLGRNVLKMDHYCPWVLRYGACSKLKLSLGNRSL-TTHLIITNKTTIEC
TP02_0446       CMYKNPGNIP-FCKWCSKFK-PDRTHHCKNCGTCVLKMDHHCPWANNCIGWRNYKYFFLTTLY-HTWLICENFTTIEF
TP02_0777       CITSNPGFIP--CEKCNSSR-PIGSHHCKTCKKCILKMDHHCVWITNCVGLCNQKYFIQFLVY-QIWAIVRGNSKIDEL
TP04_0300       CSFSNPGIIP-YCETCNIYR-PPRSVHCRLCDFCVNRFDHHCKWIGNCIGYNNYRQFIAFVFT-HSFLACTNQTTNEQ
TP04_0326       SAVTDPGVVP-YCKICNVWK-PDRTHHCSSCNRCVLNMDHHCPWIGNCVGFYNRKYFMQLLVY-HFKLVLRNSTTIEN
TP04_0459       NKLSDPGVSP-VCYTCGKTK-ALREHHCSLCNTCLLRQDHHCGWIDNCVGAGNQREFFVFLTL-IVRCMVTDVTYYEY
TP03_0510       VSLIDPVDPN-HCNVCE-YV-DPSSKHCNVCNKCITKFDHHCVWVNNCIGSVNYLYFILLLLF-HLFLIYTGQTTYQY
TP03_0623       TSFSNPGYVK-FCPTCHSYR-PPRSVHCSDCDRCIVRFDHHCPYVANCIGYYNYKIFLSFLLV-HMFIIRSNLSTYDK
cgd7_1560       TFLKNPGVII-FCSNCKIIR-PPRTVHCNICNHCVDRFDHHCPWVGTCIGAGNYKLFMLFIST-HLYIGAMNKTTYEE
cgd5_1260       LIIKDPGSLE-YCNKCSKWK-PPRAHHCTTCNICIFKMDHHCMLINNCIGYSNQKIYILFLFY-QIDYISSNSTLVES
cgd1_1380       IPDTDPGSLE-YCKWCAKYK-PDRTHHCRVCRTCVLKMDHHCPWISNCVGWGNHKHLLLLILY-HLWLVFNSMTTIEF
cgd6_4540       CTNTEPGRVP-YCKVCQVWK-PDRTHHCSECGKCVLNMDHHCPWINNCVGFYNRKFFIQLLIY-HIGFIVRNLTTIES
cgd2_2190       CTTTDPGVMP-SCAKCNNLK-PPRTHHCSVCKRCIFKMDHHCPWINNCVGINNQKHFLLFLAY-QVVCIINNTTGIEH
cgd8_4730       ISFGDPGYLK-FCDYCRMYQ-PLRTKHCTSCERCIRTHDHHCPWLGVCIGEYNRCKFWWLSLV-HLFLAYNNLTTWEN
cgd4_2080       VTAINPGESR-ECKICHLFF-EENSKHCKLCNKCIPRYDHHCKWLNTCIGEKNYRHFFLLLFF-HCYLVFRGVTTYEY
cgd7_3200       IGFENPGQVI-NCSKCNAIK-HPRTHHCSICNKCILNMDHHCPWIGQCVGLYNRKYFILFLAW-HIYLLVTNQSTIEY
cgd8_5050       SFFTDAGRVP-FCKYELVYK-PDRAHYCRQLNRNVLRMDHYCPWFGNCIGYFNYKFFFLALLY-HAYITSRNETTIEF
cgd1_820        WVLSNIGNDN-ICVTCRTER-PFRGHHCSDCGYCIQRFDHHCVWIDSCVGYGNQRAFFFFLKF-HVKSIMSNVTFYEY
ETH_00022320    AAATDPGIIP-YCSTCSIYR-PPRSVHCAVCDNCVERFDHHCPWVGNCIGRRNYRAFFAAGLD-HFSLSAYNSQSVVA
ETH_00019260    TDSRPPGEFI-RCCCCCGGV-LERSKHCRSCNKCVDVFDHHCVWINNCVGKANYRAFVGMLFA-HLYLLRHHMTTFDY
ETH_00040335    AAATDPGAVP-YCKVCNVWK-PDRTHHCSACGRCILNMDHHCPWINNCVGFYNRRFFLQLLLY-HT-----------
ETH_00032190    KTSANPGPKP-VCKWCGVCK-PDRTHHCRVCRCCILRMDHHCPWLANCVGWGNHKYFMLLLLY-HLYLLAKGMTTIEF
ETH_00004680    TFAADPGIVP-ICVTCNILK-PTRVHHCAECAHCLLRQDHHCMWVDNCIAAGNTRQFVCFLTF-TGRSILSNVTYFEF
ETH_00027035    AASTDPGVLR-FCDRCCMYQ-PLRTKHCADCGVCIRTHDHHCPWIGLLLAFNVFHRRN-RNSS--------------
```
