# Supplementary material for: Global Analysis of Apicomplexan Protein S-Acyl Transferases Reveals an Enzyme Essential for Invasion
Source: Traffic. 2013 May 29;14(8):895–911. doi: 10.1111/tra.12081 (PMC3813974; doi:10.1111/tra.12081)
Supplement: Figure S7 — Alignment of the conserved domains used for the complete phylogenetic analysis including T. gondii, N. caninum, P. falciparum and P. berghei. [file tra0014-0895-sd7.pdf]

```
                       10        20        30        40        50        60        70
               ....|....|....|....|....|....|....|....|....|....|....|....|....|....|....
PBANKA_010830  CASTDPGKVP-YCKICNVWK-PDRTHHCSACNRCVLNMDHHCPWINNCVGFYNRRFFMQLLFY-HLKLISKNSTTIEN
PBANKA_031260  CLLKNPGYLN-YCKKCNFSK-IKRAHHCSVCNKCVKKMDHHCTWINNCVGLYNQKFFILLNIY-QYWGIKTNTSGIEF
PBANKA_040200  LYFKNPGFYK-VCVTCNIIK-PPRVHHCADCFHCVVHQDHHCVWVDNCIGINNQRSFYMFILS-NTKTILTNVTFYEH
PBANKA_051200  TYNISPGYVP-YCAYSKIYK-PDRSHYCRAIDKTVLKMDHYCPWVANCIGFYNYKFFLLSLLY-HLYLTSQNYTTLEF
PBANKA_083330  SSLCNPGYIT-YCIQCDLVQ-MLRSKHCKYCKQCIKTYDHHCLWINNCVGENNRLIFFLYLYF-HTHLCMVNETTWEN
PBANKA_092730  VTSINPIDPL-ECDICGFVE-PQ-SKHCKVCNKCVSVFDHHCMWVNNCIGKKNYKYFVSLLLA-HIYLISKKMTTYEY
PBANKA_093210  CSACNPGYVD-ICDKCNYLVRPERAHHCRTCQRCVLKMDHHCPWIGTCVGEKNLKFFFLFLIY-YVYFISHNITTIES
PBANKA_124300  SIVTPPGFIP-FCKWCCKYK-PDRAHHCRICKTCILKMDHHCPWIYNCIGYNNHKYFMLSLIY-HLWLTFKNMTTIEF
PBANKA_133780  VVSSNPGFLD-LCPTCFLFK-NTRTKHCAFCDKCIDIFDHHCIFTLNCMGIDNARIFLSWILS-SIFNILENITLNEK
PBANKA_141970  TAFCDPGIIP-WCVHCNHFK-EPRSKHCYTCNNCVTKFDHHCVWLGNCIGIRNYRNFIFFILN-HLKIILLNKTTYED
PBANKA_142090  TSFMDPGIIP-YCYTCNIYR-GIRTVHCSICDNCVEKFDHHCPWVGNCIGARNYKYFIYFIFN-HIYTIVTNQTTYEQ
PF3D7_0932500  VSYRNPGYVT-YCKICDVYQ-ILRSKHCQMCKRCVRTFDHHCPWINNCVAENNRSFFLLYLYF-HSYLCLINETTWEN
PF3D7_1027900  TYKVNPGNIP-YCIHEKKYK-PDRSHYCRAIEKNVLKMDHYCPWVANCVGFYNYKFFLLSLFY-HIYLTSKNYTTLEF
PF3D7_1321400  TAFCDPGIIP-WCVNCNHFK-EPRSKHCYTCNNCVTKFDHHCVWIGNCVGNRNYRRFFFFILN-HLQMVLQNKTTYED
PF3D7_1322500  VVTSNPGYLE-LCPTCFLFK-NIRTKHCSLCDKCVEIFDHHCDFTLNCMGIENARIFLLWILL-SIMNILENITSNEK
PF3D7_0202900  CSLSDPGKIS-KCKTCNIIK-PARSKHCSYCSSCISRYDHHCFLLNNCIGGYNNMYYLVFLHI-IYFSLFNNITQNEL
PF3D7_0215900  CLCTNPGFLN-MCKKCNLLK-IKRSHHCSVCDKCIMKMDHHCFWINSCVGLYNQKYFILLNFY-QYCAIKTNTTGIEL
PF3D7_0303400  LYFKSPGFYK-ICVTCNIIK-PPRVHHCAECFHCIVHQDHHCVWVDNCIGIKNQRCFYMFIFC-NTRTILTNVTFYEH
PF3D7_0528400  SIIVSPGSIP-HCKWCCKYK-PDRTHHCRVCKSCILKMDHHCPWIYNCVGYNNHKYFMLSLIY-HIWLLINAMTTIEF
PF3D7_0609800  CASTDPGKVP-YCKICNVWK-PDRTHHCSACNRCVLNMDHHCPWINNCVGFFNRRFFIQLLFY-HLKLISKNSTTIEN
PF3D7_0714300  TSFMDPGIIP-YCYTCNIYR-GIRTVHCSICDNCVEKFDHHCPWVGNCIGARNYKYFVYFVFN-HIYTIVTNQTTYEQ
PF3D7_1115900  CSIVNPGYVD-ICDKCDFLVRPERAHHCRTCNKCILKMDHHCPWIGTCVGEKNLKFFFLFLIY-YIYFISKNITAIES
PF3D7_1121000  VTKTNPVDPL-QCDICGFVQ-PE-SKHCKVCNKCVSVFDHHCMWVNNCIGKKNYKYFVGLLST-HIFLISKKMTTYEY
TGME49_250870  AVFVSPGGVP-ECKWCLH---PDRTHHCRVCRTCVLKMDHHCPWIDNCVGWGNHKYFMLSIIY-HLYLMVKGMTTIEF
TGME49_278850  AVCTDPGRVP-YCKVCNV---PDRTHHCSACGRCVLNMDHHCPWINNCVGFYNRKYFIQLLIY-HLNLVLKNSTTIEN
TGME49_217870  CALRDPGEVS-TCTHCLR---PERAHHCSICNKCVMRMDHHCPWVGNCVGFNNYKQFLLFNFYTHLYYVLVNMTTIE-
TGME49_213550  TACSDPGILP-FCTTCNI---PPRSVHCAICDNCVERFDHHCPWLGNCIGLRNYRTFIFFVIF-HGYLIATNQTTYEQ
TGME49_224290  VAFGDPGIIP-WCTTCYF---PPRSKHCSVCNNCVRRFDHHCPWVSNCVGERNYRIFFFFLLL-NIYLIVNNRTTNEE
TGME49_224310  TAFSDPGIIP-WCTTCCL---PPRTKHCSTCDNCVQRFDHHCPWVSNCIGQRNYRVFFFFVFF-NFYLILNNLTTNED
TGME49_252200  AVVTPPGSIP-HCKWCRR---PDRAHHCRVCRQCVLKMDHHCPWIYNCVGWRNHKYFMLSLIY-HTHLVCNGMTTIEF
TGME49_255650  CSLKNPGIIN-ECSTCRF---PARSKHCRLCNVCVARFDHHCVWIGNCVGARNHGVFIIFLIT-HLYTAVWRNSTTNE
TGME49_269150  AVFTDPGSTK-QCSPCKGR--PPRAHHCKVCKECIFRMDHHCPWINNCVGLMNQKYFILFLIYSEQWEALETNTTLVE
TGME49_301370  ACVTGAGSVP-YCHKCAH---PDRAHHSRHTGTCTLKLDHYCPWVANDIGYFNYKYFYLTLLY-HTYLLSINSSTVEY
TGME49_284170  CLFSDPGAVP-SCRKCCS---PARAHHCSVCQRCILKMDHHCPWVNNCVGQTNQKFFLLFLVYFDQLSAIRANTTGIE
TGME49_229160  TVLKDPGIPR-FCRECRI---PAGSVHCDDCRVCIEGYDHHCPWTSKCVGKGNSKEFHAWIIL-------------ED
TGME49_249380  ATTTDPIDPV-ECDVCG----HERSKHCRVCNKCVDGFDHHCMWINNCVGEKNYRPFFALLVF-HIYLVRHHLTTFEY
TGME49_293730  TYSGDAGIAP-VCVTCAI---TPRIHHCADCGHCLERQDHHCVWVDTCIARNNFQPFWFFLLC-TTRVMFTNVTYYEF
TGME49_293220  TALGDPGYLK-YCEICAM---PLRTKHCGHCGRCTRTHDHHCPWIGTCVAEENRVYFYWFLFL-HTYLMLSNLTTWES
TGME49_266940  ACTVDPGRPP-LCSTCGG---PLRTHHCRICNRCVLKQDHHCPWLNQCVGLHNYRFFFLFLFFFHVYLLMGNQTTIEV
TGME49_272320  LLLSDPGIHP-LCRTCWI---PLRTKHCSICDRCVDGFDHHCVWLYNCVGSLNARLFTAWLLLGHLRNIAQNITANEV
TGME49_246650  WTGINPGYIR-FCADCRI---PLRTRHCKECDHCVLTYDHHCAFLGCCVGEFNHWRFYLFLLS-HTYLMLSNQTTWVW
NCLIV_000160   TAVGDPGYLK-FCQICFM---PLRTKHCSQCGRCTRTHDHHCPWIGTCVAEENRVYFYWFLLL-HTYLMLSNLTTWES
NCLIV_000720   VVALMPGVAT-VCVTCAI---TPRIHHCADCGHCLERQDHHCVWVDTCIARNNVQPFWFFLLS-TTRVMFTNVTYYEY
NCLIV_039120   ACAVDPGRPP-HCVKCGA---PPRSHHCRICNRCVLKQDHHCPWLNQCVGLHNYRFFFLFVFFFHVYLLLGNQTTIEM
NCLIV_030100   TVLKDPGIPR-FCRDCQI---PPGSVHCDDCRVCIEGYDHHCPWTSKCVGKGNIWEFHVWIILIFYYGLTAAFLGQEE
NCLIV_063440   WVGINPGYIR-FCIYCRV---PLRTRHCAECNHCVLTYDHHCAFLGCCIGEFNHWRFYLFLLS-HTYLILSNQTTWD-
NCLIV_065580   ASTTDPIDPI-ECDVCG----NERSKHCRVCNKCVDGFDHHCMWINNCVGDKNYRPFFVLLVA-HIYLVLHHLTTFEY
NCLIV_034970   LLLSDPGTLP-LCRTCWI---ALRTKHCFVCNRCVEGFDHHCVWVYNCVGALNARLFTSWLLVGHMRNIAQNITANEV
NCLIV_037240   AIFTDPGSTK-HCSPCRGS--PPRAHHCKVCKECIFRMDHHCPWINNCVGLMNQKYFILFLIYSEQWEALETNTTLVE
NCLIV_048280   VAFSDPGIIP-WCTTCYL---PPRSKHCSVCNNCVRRFDHHCPWVSNCVGERNYRIFFFFLVF-NIYLIANNRTTNEE
NCLIV_062610   CALRDPGEVS-TCAHCLR---PERAHHCSICNKCVMRMDHHCPWVGNCVGFNNYKQFLLFNLYTHLYYVLVNMTTIE-
NCLIV_066400   AVFVSPGGVP-ECKWCMH---PDRTHHCRVCRTCVLKMDHHCPWIDNCVGWGNHKYFMLAVIY-HLYLVVKGMTTIEF
NCLIV_067160   AVCTDPGRVP-YCKVCNV---PDRTHHCSACGRCVLNMDHHCPWINNCVGFYNRKYFIQLLIY-HLNLVLKNSTTIEN
NCLIV_068450   VAFSDPGIIP-WCTTCYL---PPRSKHCSVCNNCVRRFDHHCPWVSNCVGERNYRIFFFFLVF-DIYLIANNRTTNEE
NCLIV_069490   TACSDPGILP-FCTTCNI---PPRSVHCAICDNCVERFDHHCPWLGNCIGLRNYRTFVFFVIF-HGYLISTNQTTYEQ
NCLIV_029260   CSLKDPGVID-KCSTCLF---PARSKHCRLCNVCVARFDHHCVWIGNCVGARNHGAFIVFLVT-HIYTAVWRNSTTNE
NCLIV_028970   CLLSDPGAVP-SCRKCRS---PARAHHCSVCQRCILKMDHHCPWINNCVGQTNQKFFLLFLVYFDQLSAIRSNTTGIE
NCLIV_007310   AVITPPGSIP-HCKWCRR---PDRAHHCRVCRQCVLKMDHHCPWIYNCVGWRNHKYFMLSLIY-HTHLVCNGMTTIEF
```
