# Supplementary material for: Global Analysis of Apicomplexan Protein S-Acyl Transferases Reveals an Enzyme Essential for Invasion
Source: Traffic. 2013 May 29;14(8):895–911. doi: 10.1111/tra.12081 (PMC3813974; doi:10.1111/tra.12081)
Supplement: Table S1 — Primers used in this study for annotation of TgDHHCs. F, forward; R, reverse [file tra0014-0895-sd8.doc]

**Table S1.** Primers used in this study for annotation of TgDHHCs, F: forward, R: reverse

| **Name** | **5’-3’ sequence** |
| --- | --- |
| TgDHHC2-F1 | GAATTCTTTTCCACCTTCGCTTTCGAGCG |
| TgDHHC2-R2 | TTAATTAACGGAGGAGAAAGTGCAAAAGCTGCG |
| TgDHHC4-F1 | GGCGAATTCGTCTGCACCGCGTTTCCCG |
| TgDHHC4-R2 | GGCTTAATTAACGCCAGATGACGAGGCGCCC |
| TgDHHC4-F4 | CGAGTTGTCGGGTGACTCTGCACG |
| TgDHHC4-F6 | GCACAAGCGAACCAGCGTCAGG |
| TgDHHC4-R8 | GGCATGCATACTCCTGGTCAAATGCAGCGG |
| TgDHHC5-F1 | CGGAATTCGGAGCGACCTGGGTTCCTCGGAC |
| TgDHHC5-R2 | CCTTAATTAAGCGGCGAGTAGGGACTACTCCGGC |
| TgDHHC6-F6 | GCATCTCGTCATCTCTTGAAGG |
| TgDHHC6-R7 | CGTGAAGGAGCTCTGAAACTCCATC |
| TgDHHC7-F1 | GAATTCACCGGCCAGTCGGAGCTGC |
| TgDHHC7-R3 | TTAATTACGTCCTAAACGGATCGGAAGCCACC |
| TgDHHC9-F1 | GAATTCGCTTACAAACGATCTGCCCCTTGCC |
| TgDHHC9-R2 | TTAATTAACTCGATTTGACAAGACACCTAGTAC |
| TgDHHC11-F2 | CGTCAGAGTCCTCAGAAGAGAG |
| TgDHHC11-R3 | AACAGCAGAAGGCCTGCTGG |
| TgDHHC12-F7 | GCGAAAGGAGGCGATTGAGCG |
| TgDHHC12-R2 | CCGGCACAAAGCAGTCTCTCCAGG |
| TgDHHC13-F1 | CTCCCACTGTCTCGCGCTGG |
| TgDHHC13-F3 | GCGGTACCGTGCGTCGACGGCTTTGACCATC |
| TgDHHC13-R2 | GTCCACCTCCTCTTCGTTTCTCCCGTCTGC |
| TgDHHC14-F1 | CGAGCGCTCCACCAAAGCCCC |
| TgDHHC14-R3 | GATGAGGTCTTTGCTGCCCGGTGC |
| TgDHHC14-F4 | GTGGATCTTCCTGTGGGGCC |
| TgDHHC14-R2 | CCATACGGCGTTTGTGTCCGCC |
| TgDHHC15-F1 | CCGCCTGCTATCCCATTACTTCGTGACTTC |
| TgDHHC15-R2 | CCGGCGCAAGAAGCACAGATGGACAG |
| TgDHHC15-F3 | CCGGGTACCCACGACCATCACTGCCCCTGG |
| TgDHHC15-F6 | CTGTCCATCTGTGCTTCTTGCG |
| TgDHHC15-R7 | CGCGTGCATCTGCCGCAGTG |
| TgDHHC15-F8 | CGGGGTACCAGACTGGACCATGCTCCG |
| TgDHHC15-R9 | GGCATGCATGCTGCTTCGCTCTGTCGGAC |
| TgDHHC15-R12 | GTGTATCTACGAAGAACTCCCTC |
| TgDHHC16-F1 | GCTACACAGGAAGCCCAGGCGCGAG |
| TgDHHC16-R3 | CCTACGCACTGGTTCAGCCACGG |
| TgDHHC16-F4 | CCGGGTACCACGTGTGGGGGCGCGAAGCC |
| TgDHHC16-R6 | GGTCGCCTGCATGATCCCTCACG |
| TgDHHC17-F2 | CCGGAATTCGGAGCAGACCCCGACGCCCGCTCGG |
| TgDHHC17-R3 | CCCTACACAGTTGTAGAGCCAGACGC |
| TgDHHC17-F4 | GCGACCGATGCGTGGACGGCTTTG |
| TgDHHC17-R11 | GCGGCGGGTGTACACACTTTC |
