# Supplementary material for: Global Analysis of Apicomplexan Protein S-Acyl Transferases Reveals an Enzyme Essential for Invasion
Source: Traffic. 2013 May 29;14(8):895–911. doi: 10.1111/tra.12081 (PMC3813974; doi:10.1111/tra.12081)
Supplement: Table S2 — Primers used in this study for cloning of TgDHHCs. F, forward; R, reverse; the restriction sites are underlined [file tra0014-0895-sd9.doc]

**Table S2.** Primers used in this study for cloning of TgDHHCs.

F: forward, R: reverse, the restriction sites are underlined

| Knock-in tagging at the end of the gene for localization | |
| --- | --- |
| Name | 5’-3’ sequence |
| TgDHHC1-F5 | CCGGGTACCAATTGTGACAGAAACCGGACACACACC |
| TgDHHC1-R2 | CCGATGCATGCAACTCTTTGCCTACGTCGGCGCC |
| TgDHHC2-F3 | GGTACCGGTGCACCGGCGCAGAAAATCG |
| TgDHHC2-R4 | CCTGCAGGAAGTAAGAGTTTAGAGTGAAAAAAGTCGAAGGTCG |
| TgDHHC3-F1 | GCGGTACCTGTCGTCCTGGACGAG |
| TgDHHC3-R3 | GCTCCTGCAGGGACATGTGCATCACCGGACCC |
| TgDHHC4-F7 | CGGGGTACCCACAGACGGCAGAGATTTCACC |
| TgDHHC4-R8 | GGCATGCATACTCCTGGTCAAATGCAGCGG |
| TgDHHC5-F3 | CCGGGTACCGCGCGAGCGTATAACGGACTGC |
| TgDHHC5-R4 | GGCCCTGCAGGGAGAGATCGGTGAGACGCGCCATG |
| TgDHHC6-F8 | GCCGGTACCACGCAGGCAGAAAGACTTGG |
| TgDHHC6-R9 | CCGATGCATGGACTTGCGCGGAAAAGGAAGACC |
| TgDHHC7-F4 | CCGGGTACCAGTTCGAAAAAATGTTTATGGTCCTGTTCG |
| TgDHHC7-R5 | GGCATGCATCCACTGATGATTCAATTATTGGCTCAAAGGTC |
| TgDHHC8-F1 | GGGGTACCGCACACACGGCTACAAGACAGATAC |
| TgDHHC8-R2 | CCTGCAGGTTCGTCCTTTTTTTCTTTCTCCTCTTTTTCTCCGACTGCTGC |
| TgDHHC9-F3 | GCGGTACCGGCCCCGCGATAATATTTGCTAATGTG |
| TgDHHC9-R4 | CCGATGCATGGGTTCCGACAACGGATTTTGGCC |
| TgDHHC11-F1 | CCGGGTACCGGAAGCAGTCGCAAATGGCAGTAG |
| TgDHHC11-R2 | CGGATGCATCGGGAAGAGGCGAGAGTGGAGC |
| TgDHHC12-F5 | CGGGGTACCCGGCGAACGGAACAGAAG |
| TgDHHC12-R6 | GGCATGCATGCTCTCCGTCAGGGTGTCG |
| TgDHHC13-F3 | GCGGTACCGTGCGTCGACGGCTTTGACCATC |
| TgDHHC13-R4 | GGCATGCATGGACCTGCGACTTTTGAGTATCTTC |
| TgDHHC14-F5 | CCGGGTACCGGAGATTCGAGTCCGGTTAGCC |
| TgDHHC14-R6 | GGCATGCATCGAAGGCACCGTTGCCGGCAGC |
| TgDHHC15-F10 | GGCGGTACCGTGAGAAACGGCGACGAAGGTG |
| TgDHHC15-R13 | CCGCCTGCAGGGAGGGAGTGACAGCAGACCCGG |
| TgDHHC16-F4 | CCGGGTACCACGTGTGGGGGCGCGAAGCC |
| TgDHHC16-R5 | GCCATGCATCTCCCAAGACCTCCTCAAGAGATCGG |
| TgDHHC17-F9 | CGGGGTACCTATCTTTGGGAGCCCGTTC |
| TgDHHC17-R10 | GGCCCTGCAGGCACCACCTTCCCTTTGACG |
| Knock-in tagging upstream the DHHC motif to generate a truncated gene | |
| Name | 5’-3’ sequence |
| TgDHHC1-F11 | GGCGGGCCCAACCTCCCCCATTTGGACGGGTC |
| TgDHHC1-R12 | GGCATGCATCGCGGCGCTCGCCGGTG |
| TgDHHC2-F18 | GGCGGTACCCACGGACCGTCGCCTATGGC |
| TgDHHC2-R19 | GGCATGCATTCAGCACGCTGAGGAACAAACCAGCAC |
| TgDHHC3-F3 | CCGGGTACCCCTCTTTGCCTGGATCACCTACGTG |
| TgDHHC3-R4 | CCGCCTGCAGGTTCGCATCACGCACTTGTTGC |
| TgDHHC4-F9 | ACCGGGCCCATGATCTCAAAGCACCAACACGCATG |
| TgDHHC4-R10 | GGCATGCATATCGCTCGACGCAATTATCACATATTGC |
| TgDHHC5-F5 | CGGGGTACCGCAGTCCGCAGCCTTGAGG |
| TgDHHC5-R6 | GGCATGCATCGCCGTTGATTGTGATGTACTTGAC |
| TgDHHC6-F10 | GCCGGTACCAGACAGCGTCGTCAGTGTATATG |
| TgDHHC6-R11 | CCGATGCATCGCTGACGCCGTTGATGACCACA |
| TgDHHC7-F22 | GGCGGTACCCGGACCTGTGACGATCCCCGC |
| TgDHHC7-R23 | GGCCCTGCAGGAGTAGAAGCAAATTTCATCGACTGAGGG |
| TgDHHC8-F3 | GGGGTACCACGCGATGATCGTGCACCCAGG |
| TgDHHC8-R4 | TGCATGCATGCTTGCTCCGGGCCGGCCTGAAAAAGAC |
| TgDHHC9-F7 | GGCGGTACCCTGATATTCAGTCCCTTGGGAATCTTCTTG |
| TgDHHC9-R8 | GGCATGCATCTACCTGACTCTGCAGGACAGGAATC |
| TgDHHC11-F7 | GGCGGTACCTTCGTCACTCTTCGTCGCG |
| TgDHHC11-R8 | GGCATGCATGGGAAGGCATCTCGAAGGCG |
| TgDHHC12-F3 | CGGGGTACCGAGATGGCGTTCGGTGAC |
| TgDHHC12-R4 | CCGGCCTGCAGGACTCCACGCAAAGTTCTCATAC |
| TgDHHC13-F5 | GCCGGTACCTCGCTCGGTGACAGATTCCT |
| TgDHHC13-R6 | CCGATGCATCGATCGGGTCAGTCGTGGTCGC |
| TgDHHC14-F7 | CCGGGTACCGCGACACTGCATGCGCTTGGGAG |
| TgDHHC14-R8 | GGCCCTGCAGGCAGGGTTGGCGGTCCATTCAGAAGCG |
| TgDHHC15-F8 | CGGGGTACCAGACTGGACCATGCTCCG |
| TgDHHC15-R9 | GGCATGCATGCTGCTTCGCTCTGTCGGAC |
| TgDHHC16-F7 | GGCGGTACCGTTTCTCGGGCAGCTGGTCGTTTC |
| TgDHHC16-R8 | GCCATGCATGACTCACAGCTGCACAACCTTC |
| TgDHHC17-F7 | CGGGGTACCCCTGGTCTCCACCACAATCT |
| TgDHHC17-R8 | GGCCCTGCAGGCAGAGGACGGTAGATCCAGCA |
| Cloning of a cDNA second copy under the control of the tubulin promotor | |
| Name | 5’-3’ sequence |
| TgDHHC3-F11 | CCGGAATTCGCTAACAAAATGTACGCGACGAGCCGCGC |
| TgDHHC3-R3 | GCTCCTGCAGGGACATGTGCATCACCGGACCC |
| TgDHHC4-F6 | GCACAAGCGAACCAGCGTCAGG |
| TgDHHC4-R8 | GGCATGCATACTCCTGGTCAAATGCAGCGG |
| TgDHHC4-F11 | CCGCAATTGCTTTTTCGACAAAATGCAGCCTGCGTTAGCGG |
| TgDHHC4-R15 | GGCGAATTCCTGCATTTTTGCGCAAGTGACC |
| TgDHHC4-F14 | GGCAAGAAAGAGGACGACGACC |
| TgDHHC4-R12 | ACGAAGCAGCTCGAGAAGAGACG |
| TgDHHC5-F10 | CCGGAATTCCTTTTTCGACAAAATGTACAATCTCTCCGGAGCGG |
| TgDHHC5-R4 | GGCCCTGCAGGGAGAGATCGGTGAGACGCGCCATG |
| TgDHHC6-F12 | CGGAATTCCCGTTCACATGGCTGAGAG |
| TgDHHC6-R9 | CCGATGCATGGACTTGCGCGGAAAAGGAAGACC |
| TgDHHC12-F11 | CCGGAATTCGCTAACAAAATGGAGACTCTGCAGTGCGAAG |
| TgDHHC12-R6 | GGCATGCATGCTCTCCGTCAGGGTGTCG |
| TgDHHC16-F2 | CCGGAATTCTGTACAGAGAGCAGTGCTCG |
| TgDHHC16-R | GCCATGCATCTCCCAAGACCTCCTCAAGAGATCGG |
| Cloning into the DiCre vector | |
| Name | 5’-3’ sequence |
| TgDHHC7-F13 | GGCGAATTCCCTTTTTCGACAAAATGATCCCCCGGACCTGTGACG |
| TgDHHC7-R5 | GGCATGCATCCACTGATGATTCAATTATTGGCTCAAAGGTC |
| TgDHHC7-F38 | CCGGGTACCTCCGTTCCTCTAGTAGCTG |
| TgDHHC7-R33 | TCCGGGCCCAATTCAAAACGCTCAAGTGGACG |
| TgDHHC7-F34 | TCCGAGCTCGCCCACTGAGACGAGGGATCAGCAG |
| TgDHHC7-R35 | TCCGAGCTCAGATCTGAACGTCGCCATTGCTGCCTTCGAC |
