# Supplementary material for: Global Analysis of Apicomplexan Protein S-Acyl Transferases Reveals an Enzyme Essential for Invasion
Source: Traffic. 2013 May 29;14(8):895–911. doi: 10.1111/tra.12081 (PMC3813974; doi:10.1111/tra.12081)
Supplement: Table S3 — Summary of the PlasmoGEM data available for the PbDHHCs [file tra0014-0895-sd10.doc]

**Table S3.** Summary of the PlasmoGEM data available for the PbDHHCs

| PbDHHC | **Pb gene ID** | Design | PlasmoGEM Design ID | PlasmoGEM  Vector Available | PbG clone ID |
| --- | --- | --- | --- | --- | --- |
| PbDHHC3 | PBANKA_092730 | TAG | 31877 | YES | PbG01-2411g05 |
| PbDHHC3 | PBANKA_092730 | KO | 31869 | YES | PbG02_B-22c03 |
| PbDHHC4 | PBANKA_142090 | TAG | 65187 | YES | PbG01-2325c05 |
| PbDHHC4 | PBANKA_142090 | KO | 65179 | NO | PbG01-2332g08 |
| PbDHHC5 | PBANKA_133780 | TAG | 58312 | YES | PbG01-2389d06 |
| PbDHHC5 | PBANKA_133780 | KO | 58304 | YES | PbG01-2389d06 |
| PbDHHC6 | PBANKA_083330 | TAG | 27808 | YES | PbG02_A-48e04 |
| PbDHHC6 | PBANKA_083330 | KO | 27800 | YES | PbG01-2385g10 |
| PbDHHC7 | PBANKA_124300 | TAG | 52423 | YES | PbG01-2474f11 |
| PbDHHC7 | PBANKA_124300 | KO | 52415 | YES | PbG02_A-56f05 |
| PbDHHC8 | PBANKA_141970 | TAG | 64995 | YES | PbG01-2428e06 |
| PbDHHC8 | PBANKA_141970 | KO | 64987 | YES | PbG01-2428e06 |
| PbDHHC9 | PBANKA_093210 | TAG | 32579 | YES | PbG01-2467c05 |
| PbDHHC9 | PBANKA_093210 | KO | 32571 | YES | PbG01-2467c05 |
| PbDHHC10 | PBANKA_051200 | TAG | 15166 | YES | PbG02_A-56e08 |
| PbDHHC10 | PBANKA_051200 | KO | 15158 | YES | PbG01-2347f08 |
| PbDHHC11 | PBANKA_031260 | TAG | 10457 | YES | PbG01-2356c07 |
| PbDHHC11 | PBANKA_031260 | KO | 10449 | YES | PbG01-2356c07 |
