# Supplementary material for: Global Analysis of Apicomplexan Protein S-Acyl Transferases Reveals an Enzyme Essential for Invasion
Source: Traffic. 2013 May 29;14(8):895–911. doi: 10.1111/tra.12081 (PMC3813974; doi:10.1111/tra.12081)
Supplement: Table S4 — Primers used in this study to check integration of T. gondii constructs. F, forward; R, reverse. A and B are the position of the primers on the scheme of Figure S2 for the knockin strategy at the C-terminal par of the genes, of Figure S4 for the knockin strategy upstream of the DHHC motif and of Figure 5 for the knockout with the DiCre-lox system [file tra0014-0895-sd11.doc]

**Table S4.** Primers used in this study to check integration of *T. gondii* contructs.

F: forward, R: reverse, A and B are the position of the primers on the scheme of figure S2 for the knock-in strategy at the C-terminal par of the genes, of figure S4 for the knock-in strategy upstream of the DHHC motif and of figure 5 for the knock-out with the DiCre-lox system.

| Integration at the end of the gene by knock-in | |
| --- | --- |
| Name | 5’-3’ sequence |
| TgDHHC1-F11-AB | TTACGTCGCCGCCACCATGTTCG |
| TgDHHC1-R10-A | GAACTGGGATCCAGGAGACTTGAGGA |
| SAG1-3’-R-B | CAGTTTCTTTATAATGGGGC |
| TgDHHC2-F20-AB | GATGACCTGGAGAGGAAGAAGAGC |
| TgDHHC2-R2-A | TTAATTAACGGAGGAGAAAGTGCAAAAGCTGCG |
| TgDHHC3-F9-AB | CTCAGATCGCACCTGCATGTGC |
| TgDHHC3-R8-A | GAGGTAAGTATGCAAGAACAGACGACCAC |
| TgDHHC4-F17-AB | CTCTTCTCGAGCTGCTTCGTC |
| TgDHHC4-R18-A | CAGAGATCGACCTCACCATGC |
| TgDHHC5-F9-AB | GAACAGTCAGCGAACAGCATGG |
| TgDHHC5-R2-A | CCTTAATTAAGCGGCGAGTAGGGACTACTCCGGC |
| TgDHHC6-F4-AB | CTCCAACTGCATTGGGCAGCGC |
| TgDHHC6-R14-A | GCGTTCCTTGGTTTCACTTCGTATTCG |
| TgDHHC7-F24-AB | GGTAGCCTTACCTCTAATGACAGC |
| TgDHHC7-R19-A | GAGTATATCCACGGGTATGCCAACCTGCG |
| TgDHHC8-F5-AB | GGCAGGAGGTCTGCTGCAACGTG |
| TgDHHC8-R9-A | GACTCCCTTTCACTTCTTCTGCTTTG |
| TgDHHC9-F15-AB | CAGGCTGCGCGTGCACTATCAGC |
| TgDHHC9-R2-A | TTAATTAACTCGATTTGACAAGACACCTAGTAC |
| TgDHHC11-F4-AB | CGTCAGAGTCCTCAGAAGAGAG |
| TgDHHC11-R3-A | AACAGCAGAAGGCCTGCTGG |
| TgDHHC12-F8-AB | GAATGAGAGCGCCTGCGACCGAG |
| TgDHHC12-R2-A | CCGGCACAAAGCAGTCTCTCCAGG |
| TgDHHC13-F7-AB | CGTCTGCGGCTTCGTACACGAGC |
| TgDHHC13-R8-A | CGAGTCTCTCTACACGGCTACCGC |
| TgDHHC14-F9-AB | CTTCTAGACCCGAGCATATCCGC |
| TgDHHC14-R2-A | CCATACGGCGTTTGTGTCCGCC |
| TgDHHC15-F14-AB | GCTCTCCAACTTAACGACCTGGGAG |
| TgDHHC15-R12-A | GTGTATCTACGAAGAACTCCCTC |
| TgDHHC16-F2-AB | CCGGAATTCTGTACAGAGAGCAGTGCTCG |
| TgDHHC16-R3-A | CCTACGCACTGGTTCAGCCACGG |
| TgDHHC17-F13-AB | GGGTATTCCGTTTTATTGTCTCCAC |
| TgDHHC17-R11 | GCGGCGGGTGTACACACTTTC |
| Integration by knock-in upstream of the DHHC motif | |
| Name | 5’-3’ sequence |
| TgDHHC1-F9-AB | CTTCCCTTCTCAGTTCGCATTCCATCCGC |
| TgDHHC1-R12-A | CATCCGACGCAATTGTCAATCCACG |
| SAG1-3’-R-B | CAGTTTCTTTATAATGGGGC |
| TgDHHC3-F7-AB | GAGTCTGTGTGCGGCGTTCCTCTCG |
| TgDHHC3-R10-A | CGAGGAGGCGCCCATGAAAGTGC |
| TgDHHC4-F16-AB | CCTCTAACAGGAGCGGTGAG |
| TgDHHC4-R13-A | GAAGGTGAACACCGAAAGGAGGG |
| TgDHHC6-F1-AB | CAATTGCGTTCTGTTCGGCGTTTCTCGCTTC |
| TgDHHC6-R14-A | GCGTTCCTTGGTTTCACTTCGTATTCG |
| TgDHHC8-F7-AB | CGACACTGGCTTGTCTCGAAGC |
| TgDHHC8-R8-A | GAGATCTACGGAGGGACTTGAC |
| TgDHHC11-F5-AB | GAGAAGGAATAGCGAGACGAGGAG |
| TgDHHC11-R6 | CTGAGGACTCTGACGGCGCAG |
| TgDHHC12-F1-AB | GGTTCTGCCTCGCTCGAAGGAGC |
| TgDHHC12-R9-A | ACGCGCATCTCGCGCGTCTCTG |
| TgDHHC13-F1-AB | CTCCCACTGTCTCGCGCTGG |
| TgDHHC13-R9-A | CGGAGCAGGCGCAGACGAGAACGGC |
| TgDHHC15-F6-AB | CTGTCCATCTGTGCTTCTTGCG |
| TgDHHC15-R7-A | CGCGTGCATCTGCCGCAGTG |
| TgDHHC16-F2-AB | CCGGAATTCTGTACAGAGAGCAGTGCTCG |
| TgDHHC16-R3-A | CCTACGCACTGGTTCAGCCACGG |
| TgDHHC17-F12-AB | GAGGCTTTTCCTCGTGTTTCAGAG |
| TgDHHC17-R3-A | CCCTACACAGTTGTAGAGCCAGACGC |
| Integration of DiCre-DHHC7 construct | |
| Name | 5’-3’ sequence |
| TgDHHC7-F26-A | TCCGGGCCCTCTTTGAGCAGGGAGATGACAGC |
| Tub-prom-R1-A | CCGGAATTCAAGAAAAAATGCCAACGAGTAGTTTTC |
| TgDHFR-R8-B | GCCCACGACAGCAGACAACTTTCC |
| TgDHHC7-F29-B | CCGTCTAGAAGTTCCGTGGTATTGGTGGAC |
| TgDHHC7-F32-CD | GACATTTGCCGCCAGGTC |
| TgDHHC7-R31-C | GGCCTCGAGCTACTGCAGTAACACAGACGACAAACG |
| TgDHHC7-R23-D | GGCCCTGCAGGAGTAGAAGCAAATTTCATCGACTGAGGG |
