# Supplementary material for: Global Analysis of Apicomplexan Protein S-Acyl Transferases Reveals an Enzyme Essential for Invasion
Source: Traffic. 2013 May 29;14(8):895–911. doi: 10.1111/tra.12081 (PMC3813974; doi:10.1111/tra.12081)
Supplement: Table S5 — Primers used in this study to check integration of P. berghei constructs. The position of the primers is shown on the scheme of Figure S3 [file tra0014-0895-sd12.doc]

**Table S5.** Primers used in this study to check integration of *P. berghei* contructs. The position of the primers is shown on the scheme of figure S3

| Integration of the 3 HA epitope tag | |
| --- | --- |
| Name | 5’-3’ sequence |
| PbDHHC3-QCR1 | GCGTATGCTCTTTCCCCAAT |
| PbDHHC3-QCR2 | ACTGCTTAATGAGGTTGCGCA |
| PbDHHC4-QCR1 | TGCCTTTTAGCGAATCTCAACA |
| PbDHHC4-QCR2 | AAACGGAAACAGGTATGCAT |
| PbDHHC5-QCR1 | TGAGCCCGTTGTTGTACGAA |
| PbDHHC5-QCR2 | TTGGCTTAGTTGAAAGCGAA |
| PbDHHC6-QCR1 | AATCGGCATAAATTTGGGGA |
| PbDHHC6-QCR2 | TGTTGGGCTTGGGTCAAATGT |
| PbDHHC7-QCR1 | TCAGTAAAGGAAACAGCAAGGA |
| PbDHHC7-QCR2 | AATGTGCATGCATGGTCACA |
| PbDHHC8-QCR1 | TCTTTCTCTCTCCCACCAGCA |
| PbDHHC8-QCR2 | ACACCGCGATGTTTAAATGGT |
| PbDHHC9-QCR1 | TGGTTTTTCCCGTTCAATCCTGACA |
| PbDHHC9-QCR2 | AGCACCCCATGGCAAATAAA |
| PbDHHC10-QCR1 | TCATGTGTGCATGCGGGGTT |
| PbDHHC10-QCR2 | ACGAGGCGCCAATTGATATGT |
| PbDHHC11-QCR1 | TGCTTGTCGATCAGTATTGGGGA |
| PbDHHC11-QCR2 | TGCATATGTAACTCGTGGTGA |
| Integration of the knock out | |
| Name | 5’-3’ sequence |
| PbDHHC3-QCR1 | ACAACTCTTTGGGTTGCACA |
| PbDHHC3-QCR2 | TGGGGAAAGAGCATACGCTT |
| PbDHHC4-QCR1 | ACTGAACCGAAAAAGGAAGGA |
| PbDHHC4-QCR2 | AGGGCAAATGCTGTAAATAAGTCGA |
| PbDHHC5-QCR1 | TGAGCCCGTTGTTGTACGAA |
| PbDHHC5-QCR2 | TTGGCTTAGTTGAAAGCGAA |
| PbDHHC6-QCR1 | TCCAATGTGACCTTGTGCAG |
| PbDHHC6-QCR2 | ACCATACAAAGGTGGGTATGA |
| PbDHHC7-QCR1 | TGGAGAAACCCTAAACTCGTTCCT |
| PbDHHC7-QCR2 | AGCAGCATATTCCTTGCTGTTTCCT |
| PbDHHC8-QCR1 | TCTTTCTCTCTCCCACCAGCA |
| PbDHHC8-QCR2 | ACACCGCGATGTTTAAATGGT |
| PbDHHC9-QCR1 | TGGTTTTTCCCGTTCAATCCTGACA |
| PbDHHC9-QCR2 | AGCACCCCATGGCAAATAAA |
| PbDHHC10-QCR1 | TCCTGGAAATTGTTTTATCGGCTGT |
| PbDHHC10-QCR2 | CCCATTCCCTTTGGGCTTTCCT |
| PbDHHC11-QCR1 | TGCTTGTCGATCAGTATTGGGGA |
| PbDHHC11-QCR2 | TGCATATGTAACTCGTGGTGA |
| Genotyping for the 3 HA tag | |
| Name | 5’-3’ sequence |
| PbDHHC3 | AGAGCAAGCACACCAGCAAA |
| PbDHHC4 | TCGAACCTGCTTTCGTGCTCT |
| PbDHHC5 | TGGGCGGTTTTTCTGAACAACGT |
| PbDHHC6 | TCGCGCATACCAATGTGTATGCA |
| PbDHHC7 | TCCCAAATACCGAAGAGTGGGTGT |
| PbDHHC8 | AATTCTAGTGTCGCGGTGCT |
| PbDHHC9 | AGGCGAATATGCATGTGTGTGT |
| PbDHHC10 | ACCTGCTTTCATGTGTTCCTAGGTG |
| PbDHHC11 | AGCTTTGACAAGAATGGATGTTGT |
| Genotyping for knock-out | |
| Name | 5’-3’ sequence |
| PbDHHC3 | ACTCAACGCGCTGAGATGGA |
| PbDHHC4 | GCTGAAGAGTGTACGTTTGGGTGCA |
| PbDHHC5 | TGGGCGGTTTTTCTGAACAACGT |
| PbDHHC6 | AGCCAAACTGTTGTCGAAGT |
| PbDHHC7 | ATGCATGCCACAACTGTGTT |
| PbDHHC8 | AATTCTAGTGTCGCGGTGCT |
| PbDHHC9 | AGGCGAATATGCATGTGTGTGT |
| PbDHHC10 | TCCATTTCCACATCCAACATCCACA |
| PbDHHC11 | AGCTTTGACAAGAATGGATGTTGT |
