# Supplementary material for: Global Analysis of Apicomplexan Protein S-Acyl Transferases Reveals an Enzyme Essential for Invasion
Source: Traffic. 2013 May 29;14(8):895–911. doi: 10.1111/tra.12081 (PMC3813974; doi:10.1111/tra.12081)
Supplement: Appendix S1 — Supplemental experimental procedures [file tra0014-0895-sd13.doc]

Global analysis of apicomplexan protein S-acyl transferases

reveals an enzyme essential for invasion

Karine Frénal^1^, Chwen L. Tay^2^, Christina Mueller^1^, Ellen S. Bushell^2^, Yonggen Jia^1^, Arnault Graindorge^1^, Oliver Billker^2^, Julian C. Rayner ^2§^, and Dominique Soldati-Favre^1§^

^1^Department of Microbiology and Molecular Medicine, CMU, University of Geneva, Rue Michel-Servet 1, CH-1211 Geneva 4, Switzerland

^2^Malaria Programme, Wellcome Trust Sanger Institute, Wellcome Trust Genome Campus, Hinxton, Cambridge CB10 1SA, United Kingdom.

Supplemental Information

**Figure S1**

**Figure S2**

**Figure S3**

**Figure S4**

**Figure S5**

**Supplemental figure legends**

**Figure S1. Evidence of the expression of 16 DHHCs by the tachyzoite stage of *T. gondii* and analysis of the conservation across the phylum *Apicomplexa***

A. Cell cycle profile based on the transcriptomics across the intracellular tachyzoite cell cycle (strain RH) provided on ToxoDB by Behnke *et al*., 2010 . For comparison, ROP17 and GAP40, typical proteins of the rhoptries and IMC organelles, respectively have been added. B. Phylogenetic tree of apicomplexans DHHC-containing proteins based on neighbour-joining (NJ) distance analysis on one hand and on maximum likelihood (ML) on the other hand. Only nodes supported by a bootstrap value >80 are indicated and values >95 were considered as significant allowing to cluster sequences (colored boxes). Protein accession numbers are given according to the EuPathDB website . Sequence alignment used to compute the phylogenetic tree is presented in file S1.

**Figure S2. Genotyping of TgDHHC triple Ty tagged transgenic lines, *Pb*DHHC triple HA tagged and *Pb*DHHC KO transgenic lines**

A. Scheme of the strategy used for the C-terminal tagging of the endogenous copy of the TgDHHCs in the ΔKU80 strain, B. Genomic PCR analysis confirming the integration of the construct (amplified fragment B) and the clonality of the strains (non-amplified fragment A) for which no signal was detected by western-blot and/or immuno-fluorescence assay. C. Scheme of the strategy used for triple HA tagging and for generation of the KO cell lines showing the position of the primers used for analysis. D. Pulse field gel electrophoresis (PFGE) and Southern blot analysis of size separated *P. berghei* chromosomes using a probe specific to the *pbdhfr 3’utr*, demonstrating integration of the respective targeting vectors into the expected chromosomes.

**Figure S3. Co-localization of some *T. gondii* DHHC-containing proteins with specific markers of organelles**

A. TgDHHC1 co-localizes with GRASP-YFP, a marker of the Golgi apparatus, TgDHHC7 co-localizes with the rhoptry staining of TgARO, TgDHHC14 co-localizes with TgGAP40 staining in the growing daughter cells and TgDHHC16 staining is around the nuclear staining of ENO2. B. Aerolysin treated parasites. The staining of TgDHHC13 co-localizes with the plasma membrane marker SAG1 and not with the IMC marker GAP45.

**Figure S4. 11 DHHC-containing proteins can be individually disrupted in *T.* *gondii***

A. Scheme of the strategy used to disrupt the DHHC genes in the ΔKU80 strain. The homologous recombination takes place upstream of the DHHC motif to create a truncated and non-functional version of the protein. B. Genomic PCR analysis (or cDNA PCR analysis for TgDHHC11) confirming the integration of the constructs and the clonality of the strains. C Western-blot analysis showing that the truncated proteins TgDHHC3 and TgDHHC8 can be expressed by the parasites. D. The vesicular staining of the two truncated proteins TgDHHC3 and TgDHHC8 in the parasite is the same as the full-length corresponding proteins. Scale bar: 2 μm.

**Figure S5. 11 *T.* *gondii* DHHC-containing proteins are not critical for tachyzoite survival**

A. Plaque assay stained with GIEMSA 7 days after invasion of the host cells with ΔKU80, KI-DHHCs and KO-DHHCs. Scale bar: 0.4 mm. B. Intracellular growth assay performed by counting the parasites 24 hours after invasion of the host cells.

**Supplemental tables**

**Table S1.** Primers used in this study for annotation of TgDHHCs, F: forward, R: reverse

| **Name** | **5’-3’ sequence** |
| --- | --- |
| TgDHHC2-F1 | GAATTCTTTTCCACCTTCGCTTTCGAGCG |
| TgDHHC2-R2 | TTAATTAACGGAGGAGAAAGTGCAAAAGCTGCG |
| TgDHHC4-F1 | GGCGAATTCGTCTGCACCGCGTTTCCCG |
| TgDHHC4-R2 | GGCTTAATTAACGCCAGATGACGAGGCGCCC |
| TgDHHC4-F4 | CGAGTTGTCGGGTGACTCTGCACG |
| TgDHHC4-F6 | GCACAAGCGAACCAGCGTCAGG |
| TgDHHC4-R8 | GGCATGCATACTCCTGGTCAAATGCAGCGG |
| TgDHHC5-F1 | CGGAATTCGGAGCGACCTGGGTTCCTCGGAC |
| TgDHHC5-R2 | CCTTAATTAAGCGGCGAGTAGGGACTACTCCGGC |
| TgDHHC6-F6 | GCATCTCGTCATCTCTTGAAGG |
| TgDHHC6-R7 | CGTGAAGGAGCTCTGAAACTCCATC |
| TgDHHC7-F1 | GAATTCACCGGCCAGTCGGAGCTGC |
| TgDHHC7-R3 | TTAATTACGTCCTAAACGGATCGGAAGCCACC |
| TgDHHC9-F1 | GAATTCGCTTACAAACGATCTGCCCCTTGCC |
| TgDHHC9-R2 | TTAATTAACTCGATTTGACAAGACACCTAGTAC |
| TgDHHC11-F2 | CGTCAGAGTCCTCAGAAGAGAG |
| TgDHHC11-R3 | AACAGCAGAAGGCCTGCTGG |
| TgDHHC12-F7 | GCGAAAGGAGGCGATTGAGCG |
| TgDHHC12-R2 | CCGGCACAAAGCAGTCTCTCCAGG |
| TgDHHC13-F1 | CTCCCACTGTCTCGCGCTGG |
| TgDHHC13-F3 | GCGGTACCGTGCGTCGACGGCTTTGACCATC |
| TgDHHC13-R2 | GTCCACCTCCTCTTCGTTTCTCCCGTCTGC |
| TgDHHC14-F1 | CGAGCGCTCCACCAAAGCCCC |
| TgDHHC14-R3 | GATGAGGTCTTTGCTGCCCGGTGC |
| TgDHHC14-F4 | GTGGATCTTCCTGTGGGGCC |
| TgDHHC14-R2 | CCATACGGCGTTTGTGTCCGCC |
| TgDHHC15-F1 | CCGCCTGCTATCCCATTACTTCGTGACTTC |
| TgDHHC15-R2 | CCGGCGCAAGAAGCACAGATGGACAG |
| TgDHHC15-F3 | CCGGGTACCCACGACCATCACTGCCCCTGG |
| TgDHHC15-F6 | CTGTCCATCTGTGCTTCTTGCG |
| TgDHHC15-R7 | CGCGTGCATCTGCCGCAGTG |
| TgDHHC15-F8 | CGGGGTACCAGACTGGACCATGCTCCG |
| TgDHHC15-R9 | GGCATGCATGCTGCTTCGCTCTGTCGGAC |
| TgDHHC15-R12 | GTGTATCTACGAAGAACTCCCTC |
| TgDHHC16-F1 | GCTACACAGGAAGCCCAGGCGCGAG |
| TgDHHC16-R3 | CCTACGCACTGGTTCAGCCACGG |
| TgDHHC16-F4 | CCGGGTACCACGTGTGGGGGCGCGAAGCC |
| TgDHHC16-R6 | GGTCGCCTGCATGATCCCTCACG |
| TgDHHC17-F2 | CCGGAATTCGGAGCAGACCCCGACGCCCGCTCGG |
| TgDHHC17-R3 | CCCTACACAGTTGTAGAGCCAGACGC |
| TgDHHC17-F4 | GCGACCGATGCGTGGACGGCTTTG |
| TgDHHC17-R11 | GCGGCGGGTGTACACACTTTC |

**Table S2.** Primers used in this study for cloning of TgDHHCs.

F: forward, R: reverse, the restriction sites are underlined

| Knock-in tagging at the end of the gene for localization | |
| --- | --- |
| Name | 5’-3’ sequence |
| TgDHHC1-F5 | CCGGGTACCAATTGTGACAGAAACCGGACACACACC |
| TgDHHC1-R2 | CCGATGCATGCAACTCTTTGCCTACGTCGGCGCC |
| TgDHHC2-F3 | GGTACCGGTGCACCGGCGCAGAAAATCG |
| TgDHHC2-R4 | CCTGCAGGAAGTAAGAGTTTAGAGTGAAAAAAGTCGAAGGTCG |
| TgDHHC3-F1 | GCGGTACCTGTCGTCCTGGACGAG |
| TgDHHC3-R3 | GCTCCTGCAGGGACATGTGCATCACCGGACCC |
| TgDHHC4-F7 | CGGGGTACCCACAGACGGCAGAGATTTCACC |
| TgDHHC4-R8 | GGCATGCATACTCCTGGTCAAATGCAGCGG |
| TgDHHC5-F3 | CCGGGTACCGCGCGAGCGTATAACGGACTGC |
| TgDHHC5-R4 | GGCCCTGCAGGGAGAGATCGGTGAGACGCGCCATG |
| TgDHHC6-F8 | GCCGGTACCACGCAGGCAGAAAGACTTGG |
| TgDHHC6-R9 | CCGATGCATGGACTTGCGCGGAAAAGGAAGACC |
| TgDHHC7-F4 | CCGGGTACCAGTTCGAAAAAATGTTTATGGTCCTGTTCG |
| TgDHHC7-R5 | GGCATGCATCCACTGATGATTCAATTATTGGCTCAAAGGTC |
| TgDHHC8-F1 | GGGGTACCGCACACACGGCTACAAGACAGATAC |
| TgDHHC8-R2 | CCTGCAGGTTCGTCCTTTTTTTCTTTCTCCTCTTTTTCTCCGACTGCTGC |
| TgDHHC9-F3 | GCGGTACCGGCCCCGCGATAATATTTGCTAATGTG |
| TgDHHC9-R4 | CCGATGCATGGGTTCCGACAACGGATTTTGGCC |
| TgDHHC11-F1 | CCGGGTACCGGAAGCAGTCGCAAATGGCAGTAG |
| TgDHHC11-R2 | CGGATGCATCGGGAAGAGGCGAGAGTGGAGC |
| TgDHHC12-F5 | CGGGGTACCCGGCGAACGGAACAGAAG |
| TgDHHC12-R6 | GGCATGCATGCTCTCCGTCAGGGTGTCG |
| TgDHHC13-F3 | GCGGTACCGTGCGTCGACGGCTTTGACCATC |
| TgDHHC13-R4 | GGCATGCATGGACCTGCGACTTTTGAGTATCTTC |
| TgDHHC14-F5 | CCGGGTACCGGAGATTCGAGTCCGGTTAGCC |
| TgDHHC14-R6 | GGCATGCATCGAAGGCACCGTTGCCGGCAGC |
| TgDHHC15-F10 | GGCGGTACCGTGAGAAACGGCGACGAAGGTG |
| TgDHHC15-R13 | CCGCCTGCAGGGAGGGAGTGACAGCAGACCCGG |
| TgDHHC16-F4 | CCGGGTACCACGTGTGGGGGCGCGAAGCC |
| TgDHHC16-R5 | GCCATGCATCTCCCAAGACCTCCTCAAGAGATCGG |
| TgDHHC17-F9 | CGGGGTACCTATCTTTGGGAGCCCGTTC |
| TgDHHC17-R10 | GGCCCTGCAGGCACCACCTTCCCTTTGACG |
| Knock-in tagging upstream the DHHC motif to generate a truncated gene | |
| Name | 5’-3’ sequence |
| TgDHHC1-F11 | GGCGGGCCCAACCTCCCCCATTTGGACGGGTC |
| TgDHHC1-R12 | GGCATGCATCGCGGCGCTCGCCGGTG |
| TgDHHC2-F18 | GGCGGTACCCACGGACCGTCGCCTATGGC |
| TgDHHC2-R19 | GGCATGCATTCAGCACGCTGAGGAACAAACCAGCAC |
| TgDHHC3-F3 | CCGGGTACCCCTCTTTGCCTGGATCACCTACGTG |
| TgDHHC3-R4 | CCGCCTGCAGGTTCGCATCACGCACTTGTTGC |
| TgDHHC4-F9 | ACCGGGCCCATGATCTCAAAGCACCAACACGCATG |
| TgDHHC4-R10 | GGCATGCATATCGCTCGACGCAATTATCACATATTGC |
| TgDHHC5-F5 | CGGGGTACCGCAGTCCGCAGCCTTGAGG |
| TgDHHC5-R6 | GGCATGCATCGCCGTTGATTGTGATGTACTTGAC |
| TgDHHC6-F10 | GCCGGTACCAGACAGCGTCGTCAGTGTATATG |
| TgDHHC6-R11 | CCGATGCATCGCTGACGCCGTTGATGACCACA |
| TgDHHC7-F22 | GGCGGTACCCGGACCTGTGACGATCCCCGC |
| TgDHHC7-R23 | GGCCCTGCAGGAGTAGAAGCAAATTTCATCGACTGAGGG |
| TgDHHC8-F3 | GGGGTACCACGCGATGATCGTGCACCCAGG |
| TgDHHC8-R4 | TGCATGCATGCTTGCTCCGGGCCGGCCTGAAAAAGAC |
| TgDHHC9-F7 | GGCGGTACCCTGATATTCAGTCCCTTGGGAATCTTCTTG |
| TgDHHC9-R8 | GGCATGCATCTACCTGACTCTGCAGGACAGGAATC |
| TgDHHC11-F7 | GGCGGTACCTTCGTCACTCTTCGTCGCG |
| TgDHHC11-R8 | GGCATGCATGGGAAGGCATCTCGAAGGCG |
| TgDHHC12-F3 | CGGGGTACCGAGATGGCGTTCGGTGAC |
| TgDHHC12-R4 | CCGGCCTGCAGGACTCCACGCAAAGTTCTCATAC |
| TgDHHC13-F5 | GCCGGTACCTCGCTCGGTGACAGATTCCT |
| TgDHHC13-R6 | CCGATGCATCGATCGGGTCAGTCGTGGTCGC |
| TgDHHC14-F7 | CCGGGTACCGCGACACTGCATGCGCTTGGGAG |
| TgDHHC14-R8 | GGCCCTGCAGGCAGGGTTGGCGGTCCATTCAGAAGCG |
| TgDHHC15-F8 | CGGGGTACCAGACTGGACCATGCTCCG |
| TgDHHC15-R9 | GGCATGCATGCTGCTTCGCTCTGTCGGAC |
| TgDHHC16-F7 | GGCGGTACCGTTTCTCGGGCAGCTGGTCGTTTC |
| TgDHHC16-R8 | GCCATGCATGACTCACAGCTGCACAACCTTC |
| TgDHHC17-F7 | CGGGGTACCCCTGGTCTCCACCACAATCT |
| TgDHHC17-R8 | GGCCCTGCAGGCAGAGGACGGTAGATCCAGCA |
| Cloning of a cDNA second copy under the control of the tubulin promotor | |
| Name | 5’-3’ sequence |
| TgDHHC3-F11 | CCGGAATTCGCTAACAAAATGTACGCGACGAGCCGCGC |
| TgDHHC3-R3 | GCTCCTGCAGGGACATGTGCATCACCGGACCC |
| TgDHHC4-F6 | GCACAAGCGAACCAGCGTCAGG |
| TgDHHC4-R8 | GGCATGCATACTCCTGGTCAAATGCAGCGG |
| TgDHHC4-F11 | CCGCAATTGCTTTTTCGACAAAATGCAGCCTGCGTTAGCGG |
| TgDHHC4-R15 | GGCGAATTCCTGCATTTTTGCGCAAGTGACC |
| TgDHHC4-F14 | GGCAAGAAAGAGGACGACGACC |
| TgDHHC4-R12 | ACGAAGCAGCTCGAGAAGAGACG |
| TgDHHC5-F10 | CCGGAATTCCTTTTTCGACAAAATGTACAATCTCTCCGGAGCGG |
| TgDHHC5-R4 | GGCCCTGCAGGGAGAGATCGGTGAGACGCGCCATG |
| TgDHHC6-F12 | CGGAATTCCCGTTCACATGGCTGAGAG |
| TgDHHC6-R9 | CCGATGCATGGACTTGCGCGGAAAAGGAAGACC |
| TgDHHC12-F11 | CCGGAATTCGCTAACAAAATGGAGACTCTGCAGTGCGAAG |
| TgDHHC12-R6 | GGCATGCATGCTCTCCGTCAGGGTGTCG |
| TgDHHC16-F2 | CCGGAATTCTGTACAGAGAGCAGTGCTCG |
| TgDHHC16-R | GCCATGCATCTCCCAAGACCTCCTCAAGAGATCGG |
| Cloning into the DiCre vector | |
| Name | 5’-3’ sequence |
| TgDHHC7-F13 | GGCGAATTCCCTTTTTCGACAAAATGATCCCCCGGACCTGTGACG |
| TgDHHC7-R5 | GGCATGCATCCACTGATGATTCAATTATTGGCTCAAAGGTC |
| TgDHHC7-F38 | CCGGGTACCTCCGTTCCTCTAGTAGCTG |
| TgDHHC7-R33 | TCCGGGCCCAATTCAAAACGCTCAAGTGGACG |
| TgDHHC7-F34 | TCCGAGCTCGCCCACTGAGACGAGGGATCAGCAG |
| TgDHHC7-R35 | TCCGAGCTCAGATCTGAACGTCGCCATTGCTGCCTTCGAC |

**Table S3.** Summary of the PlasmoGEM data available for the PbDHHCs

| PbDHHC | **Pb gene ID** | Design | PlasmoGEM Design ID | PlasmoGEM  Vector Available | PbG clone ID |
| --- | --- | --- | --- | --- | --- |
| PbDHHC3 | PBANKA_092730 | TAG | 31877 | YES | PbG01-2411g05 |
| PbDHHC3 | PBANKA_092730 | KO | 31869 | YES | PbG02_B-22c03 |
| PbDHHC4 | PBANKA_142090 | TAG | 65187 | YES | PbG01-2325c05 |
| PbDHHC4 | PBANKA_142090 | KO | 65179 | NO | PbG01-2332g08 |
| PbDHHC5 | PBANKA_133780 | TAG | 58312 | YES | PbG01-2389d06 |
| PbDHHC5 | PBANKA_133780 | KO | 58304 | YES | PbG01-2389d06 |
| PbDHHC6 | PBANKA_083330 | TAG | 27808 | YES | PbG02_A-48e04 |
| PbDHHC6 | PBANKA_083330 | KO | 27800 | YES | PbG01-2385g10 |
| PbDHHC7 | PBANKA_124300 | TAG | 52423 | YES | PbG01-2474f11 |
| PbDHHC7 | PBANKA_124300 | KO | 52415 | YES | PbG02_A-56f05 |
| PbDHHC8 | PBANKA_141970 | TAG | 64995 | YES | PbG01-2428e06 |
| PbDHHC8 | PBANKA_141970 | KO | 64987 | YES | PbG01-2428e06 |
| PbDHHC9 | PBANKA_093210 | TAG | 32579 | YES | PbG01-2467c05 |
| PbDHHC9 | PBANKA_093210 | KO | 32571 | YES | PbG01-2467c05 |
| PbDHHC10 | PBANKA_051200 | TAG | 15166 | YES | PbG02_A-56e08 |
| PbDHHC10 | PBANKA_051200 | KO | 15158 | YES | PbG01-2347f08 |
| PbDHHC11 | PBANKA_031260 | TAG | 10457 | YES | PbG01-2356c07 |
| PbDHHC11 | PBANKA_031260 | KO | 10449 | YES | PbG01-2356c07 |

**Table S4.** Primers used in this study to check integration of *T. gondii* contructs.

F: forward, R: reverse, A and B are the position of the primers on the scheme of figure S2 for the knock-in strategy at the C-terminal par of the genes, of figure S4 for the knock-in strategy upstream of the DHHC motif and of figure 5 for the knock-out with the DiCre-lox system.

| Integration at the end of the gene by knock-in | |
| --- | --- |
| Name | 5’-3’ sequence |
| TgDHHC1-F11-AB | TTACGTCGCCGCCACCATGTTCG |
| TgDHHC1-R10-A | GAACTGGGATCCAGGAGACTTGAGGA |
| SAG1-3’-R-B | CAGTTTCTTTATAATGGGGC |
| TgDHHC2-F20-AB | GATGACCTGGAGAGGAAGAAGAGC |
| TgDHHC2-R2-A | TTAATTAACGGAGGAGAAAGTGCAAAAGCTGCG |
| TgDHHC3-F9-AB | CTCAGATCGCACCTGCATGTGC |
| TgDHHC3-R8-A | GAGGTAAGTATGCAAGAACAGACGACCAC |
| TgDHHC4-F17-AB | CTCTTCTCGAGCTGCTTCGTC |
| TgDHHC4-R18-A | CAGAGATCGACCTCACCATGC |
| TgDHHC5-F9-AB | GAACAGTCAGCGAACAGCATGG |
| TgDHHC5-R2-A | CCTTAATTAAGCGGCGAGTAGGGACTACTCCGGC |
| TgDHHC6-F4-AB | CTCCAACTGCATTGGGCAGCGC |
| TgDHHC6-R14-A | GCGTTCCTTGGTTTCACTTCGTATTCG |
| TgDHHC7-F24-AB | GGTAGCCTTACCTCTAATGACAGC |
| TgDHHC7-R19-A | GAGTATATCCACGGGTATGCCAACCTGCG |
| TgDHHC8-F5-AB | GGCAGGAGGTCTGCTGCAACGTG |
| TgDHHC8-R9-A | GACTCCCTTTCACTTCTTCTGCTTTG |
| TgDHHC9-F15-AB | CAGGCTGCGCGTGCACTATCAGC |
| TgDHHC9-R2-A | TTAATTAACTCGATTTGACAAGACACCTAGTAC |
| TgDHHC11-F4-AB | CGTCAGAGTCCTCAGAAGAGAG |
| TgDHHC11-R3-A | AACAGCAGAAGGCCTGCTGG |
| TgDHHC12-F8-AB | GAATGAGAGCGCCTGCGACCGAG |
| TgDHHC12-R2-A | CCGGCACAAAGCAGTCTCTCCAGG |
| TgDHHC13-F7-AB | CGTCTGCGGCTTCGTACACGAGC |
| TgDHHC13-R8-A | CGAGTCTCTCTACACGGCTACCGC |
| TgDHHC14-F9-AB | CTTCTAGACCCGAGCATATCCGC |
| TgDHHC14-R2-A | CCATACGGCGTTTGTGTCCGCC |
| TgDHHC15-F14-AB | GCTCTCCAACTTAACGACCTGGGAG |
| TgDHHC15-R12-A | GTGTATCTACGAAGAACTCCCTC |
| TgDHHC16-F2-AB | CCGGAATTCTGTACAGAGAGCAGTGCTCG |
| TgDHHC16-R3-A | CCTACGCACTGGTTCAGCCACGG |
| TgDHHC17-F13-AB | GGGTATTCCGTTTTATTGTCTCCAC |
| TgDHHC17-R11 | GCGGCGGGTGTACACACTTTC |
| Integration by knock-in upstream of the DHHC motif | |
| Name | 5’-3’ sequence |
| TgDHHC1-F9-AB | CTTCCCTTCTCAGTTCGCATTCCATCCGC |
| TgDHHC1-R12-A | CATCCGACGCAATTGTCAATCCACG |
| SAG1-3’-R-B | CAGTTTCTTTATAATGGGGC |
| TgDHHC3-F7-AB | GAGTCTGTGTGCGGCGTTCCTCTCG |
| TgDHHC3-R10-A | CGAGGAGGCGCCCATGAAAGTGC |
| TgDHHC4-F16-AB | CCTCTAACAGGAGCGGTGAG |
| TgDHHC4-R13-A | GAAGGTGAACACCGAAAGGAGGG |
| TgDHHC6-F1-AB | CAATTGCGTTCTGTTCGGCGTTTCTCGCTTC |
| TgDHHC6-R14-A | GCGTTCCTTGGTTTCACTTCGTATTCG |
| TgDHHC8-F7-AB | CGACACTGGCTTGTCTCGAAGC |
| TgDHHC8-R8-A | GAGATCTACGGAGGGACTTGAC |
| TgDHHC11-F5-AB | GAGAAGGAATAGCGAGACGAGGAG |
| TgDHHC11-R6 | CTGAGGACTCTGACGGCGCAG |
| TgDHHC12-F1-AB | GGTTCTGCCTCGCTCGAAGGAGC |
| TgDHHC12-R9-A | ACGCGCATCTCGCGCGTCTCTG |
| TgDHHC13-F1-AB | CTCCCACTGTCTCGCGCTGG |
| TgDHHC13-R9-A | CGGAGCAGGCGCAGACGAGAACGGC |
| TgDHHC15-F6-AB | CTGTCCATCTGTGCTTCTTGCG |
| TgDHHC15-R7-A | CGCGTGCATCTGCCGCAGTG |
| TgDHHC16-F2-AB | CCGGAATTCTGTACAGAGAGCAGTGCTCG |
| TgDHHC16-R3-A | CCTACGCACTGGTTCAGCCACGG |
| TgDHHC17-F12-AB | GAGGCTTTTCCTCGTGTTTCAGAG |
| TgDHHC17-R3-A | CCCTACACAGTTGTAGAGCCAGACGC |
| Integration of DiCre-DHHC7 construct | |
| Name | 5’-3’ sequence |
| TgDHHC7-F26-A | TCCGGGCCCTCTTTGAGCAGGGAGATGACAGC |
| Tub-prom-R1-A | CCGGAATTCAAGAAAAAATGCCAACGAGTAGTTTTC |
| TgDHFR-R8-B | GCCCACGACAGCAGACAACTTTCC |
| TgDHHC7-F29-B | CCGTCTAGAAGTTCCGTGGTATTGGTGGAC |
| TgDHHC7-F32-CD | GACATTTGCCGCCAGGTC |
| TgDHHC7-R31-C | GGCCTCGAGCTACTGCAGTAACACAGACGACAAACG |
| TgDHHC7-R23-D | GGCCCTGCAGGAGTAGAAGCAAATTTCATCGACTGAGGG |

**Table S5.** Primers used in this study to check integration of *P. berghei* contructs. The position of the primers is shown on the scheme of figure S3

| Integration of the 3 HA epitope tag | |
| --- | --- |
| Name | 5’-3’ sequence |
| PbDHHC3-QCR1 | GCGTATGCTCTTTCCCCAAT |
| PbDHHC3-QCR2 | ACTGCTTAATGAGGTTGCGCA |
| PbDHHC4-QCR1 | TGCCTTTTAGCGAATCTCAACA |
| PbDHHC4-QCR2 | AAACGGAAACAGGTATGCAT |
| PbDHHC5-QCR1 | TGAGCCCGTTGTTGTACGAA |
| PbDHHC5-QCR2 | TTGGCTTAGTTGAAAGCGAA |
| PbDHHC6-QCR1 | AATCGGCATAAATTTGGGGA |
| PbDHHC6-QCR2 | TGTTGGGCTTGGGTCAAATGT |
| PbDHHC7-QCR1 | TCAGTAAAGGAAACAGCAAGGA |
| PbDHHC7-QCR2 | AATGTGCATGCATGGTCACA |
| PbDHHC8-QCR1 | TCTTTCTCTCTCCCACCAGCA |
| PbDHHC8-QCR2 | ACACCGCGATGTTTAAATGGT |
| PbDHHC9-QCR1 | TGGTTTTTCCCGTTCAATCCTGACA |
| PbDHHC9-QCR2 | AGCACCCCATGGCAAATAAA |
| PbDHHC10-QCR1 | TCATGTGTGCATGCGGGGTT |
| PbDHHC10-QCR2 | ACGAGGCGCCAATTGATATGT |
| PbDHHC11-QCR1 | TGCTTGTCGATCAGTATTGGGGA |
| PbDHHC11-QCR2 | TGCATATGTAACTCGTGGTGA |
| Integration of the knock out | |
| Name | 5’-3’ sequence |
| PbDHHC3-QCR1 | ACAACTCTTTGGGTTGCACA |
| PbDHHC3-QCR2 | TGGGGAAAGAGCATACGCTT |
| PbDHHC4-QCR1 | ACTGAACCGAAAAAGGAAGGA |
| PbDHHC4-QCR2 | AGGGCAAATGCTGTAAATAAGTCGA |
| PbDHHC5-QCR1 | TGAGCCCGTTGTTGTACGAA |
| PbDHHC5-QCR2 | TTGGCTTAGTTGAAAGCGAA |
| PbDHHC6-QCR1 | TCCAATGTGACCTTGTGCAG |
| PbDHHC6-QCR2 | ACCATACAAAGGTGGGTATGA |
| PbDHHC7-QCR1 | TGGAGAAACCCTAAACTCGTTCCT |
| PbDHHC7-QCR2 | AGCAGCATATTCCTTGCTGTTTCCT |
| PbDHHC8-QCR1 | TCTTTCTCTCTCCCACCAGCA |
| PbDHHC8-QCR2 | ACACCGCGATGTTTAAATGGT |
| PbDHHC9-QCR1 | TGGTTTTTCCCGTTCAATCCTGACA |
| PbDHHC9-QCR2 | AGCACCCCATGGCAAATAAA |
| PbDHHC10-QCR1 | TCCTGGAAATTGTTTTATCGGCTGT |
| PbDHHC10-QCR2 | CCCATTCCCTTTGGGCTTTCCT |
| PbDHHC11-QCR1 | TGCTTGTCGATCAGTATTGGGGA |
| PbDHHC11-QCR2 | TGCATATGTAACTCGTGGTGA |
| Genotyping for the 3 HA tag | |
| Name | 5’-3’ sequence |
| PbDHHC3 | AGAGCAAGCACACCAGCAAA |
| PbDHHC4 | TCGAACCTGCTTTCGTGCTCT |
| PbDHHC5 | TGGGCGGTTTTTCTGAACAACGT |
| PbDHHC6 | TCGCGCATACCAATGTGTATGCA |
| PbDHHC7 | TCCCAAATACCGAAGAGTGGGTGT |
| PbDHHC8 | AATTCTAGTGTCGCGGTGCT |
| PbDHHC9 | AGGCGAATATGCATGTGTGTGT |
| PbDHHC10 | ACCTGCTTTCATGTGTTCCTAGGTG |
| PbDHHC11 | AGCTTTGACAAGAATGGATGTTGT |
| Genotyping for knock-out | |
| Name | 5’-3’ sequence |
| PbDHHC3 | ACTCAACGCGCTGAGATGGA |
| PbDHHC4 | GCTGAAGAGTGTACGTTTGGGTGCA |
| PbDHHC5 | TGGGCGGTTTTTCTGAACAACGT |
| PbDHHC6 | AGCCAAACTGTTGTCGAAGT |
| PbDHHC7 | ATGCATGCCACAACTGTGTT |
| PbDHHC8 | AATTCTAGTGTCGCGGTGCT |
| PbDHHC9 | AGGCGAATATGCATGTGTGTGT |
| PbDHHC10 | TCCATTTCCACATCCAACATCCACA |
| PbDHHC11 | AGCTTTGACAAGAATGGATGTTGT |

**Supplemental experimental procedures**

# *Activation and use of Aeromonas hydrophila aerolysin*

Prior to use, purified recombinant protoxin was activated for 15 min at 37°C in 100 µl of PBS with 4 µl of trypsin diluted at 1 mg/ml into HBS (140 mM NaCl, 2.7 mM KCl, 20 mM Hepes, pH 7.4). For experiments, freshly harvested parasites were washed with PBS and incubated for 10 min at 37°C on coverslips coated with poly-L-lysine. Parasites were then treated with aerolysin at 80-100 ng/ml for 4 hr at 37°C before fixation.

*T. gondii plaque assay*

Host cells were infected with parasites for 7 days before fixation with PFA/GA. Giemsa staining was then performed as described in Plattner *et al.*, 2008 .

*T. gondii intracellular growth assay*

HFF cells were inoculated with parasites allowed to grow for 24 hr before fixation with PFA/GA. Double IFA were performed using α-GAP45 and α-actin. The number of parasites per vacuole was determined by counting the parasites in 100 vacuoles in duplicate and for three independent experiments.

**Supplemental references**

1. Gajria B, Bahl A, Brestelli J, Dommer J, Fischer S, Gao X, Heiges M, Iodice J, Kissinger JC, Mackey AJ, Pinney DF, Roos DS, Stoeckert CJ, Jr., Wang H, Brunk BP. ToxoDB: an integrated *Toxoplasma gondii* database resource. Nucleic acids research 2008;36(Database issue):D553-556.

2. Behnke MS, Wootton JC, Lehmann MM, Radke JB, Lucas O, Nawas J, Sibley LD, White MW. Coordinated progression through two subtranscriptomes underlies the tachyzoite cycle of *Toxoplasma gondii*. PLoS One 2010;5(8):e12354.

3. Aurrecoechea C, Heiges M, Wang H, Wang Z, Fischer S, Rhodes P, Miller J, Kraemer E, Stoeckert CJ, Jr., Roos DS, Kissinger JC. ApiDB: integrated resources for the apicomplexan bioinformatics resource center. Nucleic acids research 2007;35(Database issue):D427-430.

4. Plattner F, Yarovinsky F, Romero S, Didry D, Carlier MF, Sher A, Soldati-Favre D. *Toxoplasma* profilin is essential for host cell invasion and TLR11-dependent induction of an interleukin-12 response. Cell Host Microbe 2008;3(2):77-87.

**Supplemental files**

**File S1.** Alignment of the conserved domains used for the phylogenetic analysis
